# The mitochondrial genome of the ascalaphid owlfly *Libelloides macaronius *and comparative evolutionary mitochondriomics of neuropterid insects

**DOI:** 10.1186/1471-2164-12-221

**Published:** 2011-05-10

**Authors:** Enrico Negrisolo, Massimiliano Babbucci, Tomaso Patarnello

**Affiliations:** 1 Department of Public Health, Comparative Pathology and Veterinary Hygiene, University of Padova, Agripolis, Viale dell'Università 16, 35020 Legnaro, Italy

## Abstract

**Background:**

The insect order Neuroptera encompasses more than 5,700 described species. To date, only three neuropteran mitochondrial genomes have been fully and one partly sequenced. Current knowledge on neuropteran mitochondrial genomes is limited, and new data are strongly required. In the present work, the mitochondrial genome of the ascalaphid owlfly *Libelloides macaronius *is described and compared with the known neuropterid mitochondrial genomes: Megaloptera, Neuroptera and Raphidioptera. These analyses are further extended to other endopterygotan orders.

**Results:**

The mitochondrial genome of *L. macaronius *is a circular molecule 15,890 bp long. It includes the entire set of 37 genes usually present in animal mitochondrial genomes. The gene order of this newly sequenced genome is unique among Neuroptera and differs from the ancestral type of insects in the translocation of *trnC*. The *L. macaronius *genome shows the lowest A+T content (74.50%) among known neuropterid genomes. Protein-coding genes possess the typical mitochondrial start codons, except for *cox1*, which has an unusual ACG. Comparisons among endopterygotan mitochondrial genomes showed that A+T content and AT/GC-skews exhibit a broad range of variation among 84 analyzed taxa. Comparative analyses showed that neuropterid mitochondrial protein-coding genes experienced complex evolutionary histories, involving features ranging from codon usage to rate of substitution, that make them potential markers for population genetics/phylogenetics studies at different taxonomic ranks. The 22 tRNAs show variable substitution patterns in Neuropterida, with higher sequence conservation in genes located on the α strand. Inferred secondary structures for neuropterid *rrnS *and *rrnL *genes largely agree with those known for other insects. For the first time, a model is provided for domain I of an insect *rrnL*. The control region in Neuropterida, as in other insects, is fast-evolving genomic region, characterized by AT-rich motifs.

**Conclusions:**

The new genome shares many features with known neuropteran genomes but differs in its low A+T content. Comparative analysis of neuropterid mitochondrial genes showed that they experienced distinct evolutionary patterns. Both tRNA families and ribosomal RNAs show composite substitution pathways. The neuropterid mitochondrial genome is characterized by a complex evolutionary history.

## Background

**Figure 1:**
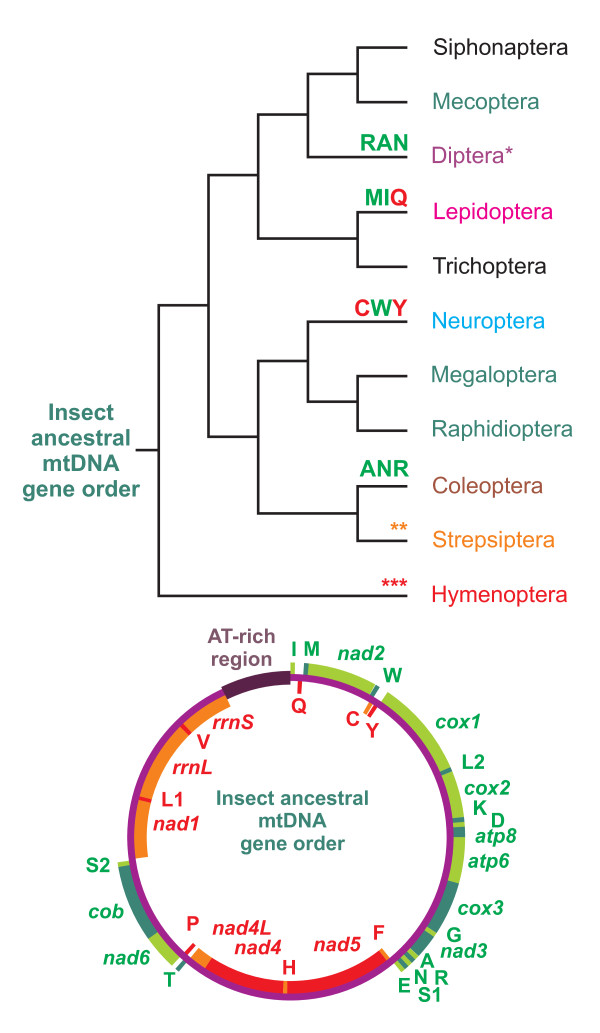
**Phylogenetic relationships among holometabolous orders and mtDNA gene order distribution**. The phylogenetic relationships among holometabolous orders are based on the work of Wiegmann et al. [53]. Black name, order for which there is no available full-length mtDNA; dark-green name, order exhibiting the insect ancestral gene order; variously colored name, order that has a gene order different from the insect ancestral gene order. **, ***, several distinct gene orders differing from the insect ancestral gene order. In the case of Diptera, only the family Culicidae has a gene order different from the ancestral arrangement. In the case of Coleoptera the gene order change is restricted to *Mordella atrata *[14]. Genes coded on the α strand (clockwise orientation) are light/deep-green. Genes coded on the β strand (counterclockwise orientation) are red or orange. Alternation of colors was applied for clarity. Gene names are the standard abbreviations used in this paper; tRNA genes are indicated by the single-letter IUPAC-IUB abbreviation for their corresponding amino acid in the figure. On the branch leading to the relative holometabolous order, the genomic change characterizing the gene order is shown. In case of multiple gene orders within a single order, the changes were not depicted to avoid overcrowding.

Insect mitochondrial genomes (mtDNAs) are usually a double-strand circular molecule containing 13 protein-coding genes (PCGs), 22 transfer RNAs (tRNAs) and 2 ribosomal RNAs, i.e., the small and large subunits [1]. A notable exception is represented by the mtDNA of the human body louse *Pediculus humanus*, which consists of 18 miniature circular chromosomes containing one to three genes [2]. Insect mtDNA size is typically in the range of 14 to 20 kbp, but genomes exceeding these values are known [3]. In these latter cases, the size increase is connected to the expansion of the main non-coding region, named the AT-rich or control region [3]. The gene order is a feature of mtDNA that can provide important evidence to establish evolutionary relationships among taxa at high and/or low taxonomic levels [4,5]. The most widespread gene order in insect mtDNAs is shown in Figure 1. This gene order, initially determined for *Drosophila yakuba *mtDNA, is considered ancestral for the entire class Insecta [5-7]. Several gene orders departing from the ancestral arrangement exist and can be restricted to single species as in the strepsipteran *Mengenilla australiensis *or common to whole groups of higher taxonomic rank, ranging from family (e.g., Culicidae) to order (e.g., Lepidoptera) (Figure 1) [8-12]. In the latter cases, the peculiar gene order becomes an important marker to delimit taxonomic boundaries and constitutes a major signature of mtDNA. To date, full-length mtDNAs in insects have been sequenced in an imbalanced manner, with whole orders still lacking any published information or being poorly represented in data banks. In this respect, the neuropterid orders Megaloptera, Neuroptera (also named Planipennia), and Raphidioptera are underrepresented taxa. Only recently have sequences become available for these taxa [13-15]. Currently, full-length mtDNAs are known for the megalopterans *Corydalus cornutus *and *Protohermes concolorus*, both members of the Corydalidae family, and *Sialis hamata*, of the Sialidae [13-15]. Three mtDNAs are available for the neuropterans *Ascaloptynx appendiculatus *(Ascalaphidae), *Ditaxis latistyla *(Mantispidae), and *Polystoechotes punctatus *(Polystoechotidae) [13,14]. A complete mtDNA sequence exists for the raphidiopteran *Mongoloraphidia harmandi *(Raphidiidae) [14]. Finally, partial mtDNA sequences are available for the neuropteran *Myrmelon immaculatus *(Myrmelontidae) and the raphidiopteran *Agulla *sp. (Raphidiidae) [13].

Here we determined the complete mtDNA sequence of the ascalaphid owlfly *Libelloides macaronius *(Scopoli, 1763) (Neuroptera, Ascalaphidae). The newly determined genome was compared with available neuropterid mtDNAs (complete and partial) as well as with genomes obtained from other holometabolous insects (Table 1) [6,9-52]. The analyses were performed following a comparative and evolutionary perspective.

**Table 1:** List of endopterygotan taxa analized in present paper

ORD	TAXON	ACN	REF	ORD	TAXON	ACN	REF
COL	*Acmaeodera *sp.	FJ613420	[16]	DIP	*Drosophila simulans*	AF200833	[31]
COL	*Adelium *sp	FJ613422	[16]	DIP	*Drosophila yakuba*	X03240	[6]
COL	*Anoplophora glabripennis*	DQ768215	UNP	DIP	*Haematobia irritans*	DQ029097	[33]
COL	*Apatides fortis*	FJ613421	[16]	DIP	*Lucilia sericata*	AJ422212	[34]
COL	*Chaetosoma scaritides*	EU877951	[17]	DIP	*Mayetiola destructor*	GQ387648	[35]
COL	*Chauliognathus opacus*	FJ613418	[16]	DIP	*Rhopalomyia pomum*	GQ387649	[35]
COL	*Chrysochroa fulgidissima*	EU826485	[18]	DIP	*Simosyrphus grandicornis*	DQ866050	[30]
COL	*Crioceris duodecimpunctata*	AF467886	[19]	DIP	*Trichophthalma punctata*	DQ866051	[30]
COL	*Cyphon *sp.	EU877949	[17]	HYM	*Abispa ephippium*	EU302588	[36]
COL	*Hydroscapha granulum*	AM493667	UNP	HYM	*Apis mellifera ligustica*	L06178	[37]
COL	*Lucanus mazama*	FJ613419	[16]	HYM	*Bombus ignitus*	DQ870926	[38]
COL	*Macrogyrus oblongus*	FJ859901	[14]	HYM	*Cephus cinctus*	FJ478173	[39]
COL	*Mordella atrata*	FJ859904	[14]	HYM	*Diadegma semiclausum*	EU871947	[40]
COL	*Priasilpha obscura*	EU877952	[17]	HYM	*Evania appendigaster*	FJ593187	[41]
COL	*Psacothea hilaris*	FJ424074	[20]	HYM	*Melipona bicolor*	AF466146	[42]
COL	*Pyrocoelia rufa*	AF452048	[21]	HYM	*Orussus occidentalis*	FJ478174	[39]
COL	*Pyrophorus divergens*	EF398270	[22]	HYM	*Vanhornia eucnemidarum*	DQ302100	[39]
COL	*Rhagophthalmus lufengensis*	DQ888607	[23]	LEP	*Acraea issoria*	GQ376195	[43]
COL	*Rhagophthalmus ohbai*	AB267275	[23]	LEP	*Adoxophyes honmai*	DQ073916	[44]
COL	*Rhopaea magnicornis*	FJ859903	[14]	LEP	*Antheraea pernyi*	AY242996	[45]
COL	*Sphaerius *sp.	EU877950	[17]	LEP	*Antheraea yamamai*	EU726630	[46]
COL	*Tetraphalerus bruchi*	EU877953	[17]	LEP	*Artogeia melete*	EU597124	[47]
COL	*Trachypachus holmbergi*	EU877954	[17]	LEP	*Bombyx mandarina*	AB070263	[48]
COL	*Tribolium castaneum*	AJ312413	[24]	LEP	*Bombyx mori*	AF149768	[48]
DIP	*Aedes aegypti*	EU352212	UNP	LEP	*Coreana raphaelis*	DQ102703	[49]
DIP	*Aedes albopictus*	AY072044	UNP	LEP	*Diatraea saccharalis*	FJ240227	UNP
DIP	*Anopheles gambiae*	L20934	[9]	LEP	*Eriogyna pyretorum*	FJ685653	[50]
DIP	*Anopheles quadrimaculatus A*	L04272	[10]	LEP	*Lymantria dispar*	FJ617240	UNP
DIP	*Bactrocera carambolae*	EF014414	UNP	LEP	*Manduca sexta*	EU286785	[11]
DIP	*Bactrocera dorsalis*	DQ845759	[25]	LEP	*Ochrogaster lunifer*	AM946601	[12]
DIP	*Bactrocera oleae*	AY210702	[26]	LEP	*Phthonandria atrilineata*	EU569764	[51]
DIP	*Bactrocera papayae*	DQ917578	UNP	LEP	*Saturnia boisduvalii*	EF622227	[52]
DIP	*Bactrocera philippinensis*	DQ995281	UNP	NEU	*Ascaloptynx appendiculatus*	FJ171324	[13]
DIP	*Ceratitis capitata*	AJ242872	[27]	NEU	*Ditaxis biseriata*	FJ859906	[14]
DIP	*Chrysomya putoria*	AF352790	[28]	**NEU**	***Libelloides macaronius***	**FR669150**	[**]
DIP	*Cochliomyia hominivorax*	AF260826	[29]	NEU	*Myrmelon immaculatus*	FJ207458-9	[13]
DIP	*Culicoides arakawae*	AB361004	UNP	NEU	*Polystoechotes punctatus*	FJ171325	[13]
DIP	*Cydistomyia duplonotata*	DQ866052	[30]	MEG	*Corydalus cornutus*	FJ171323	[13]
DIP	*Dermatobia hominis*	AY463155	UNP	MEG	*Protohermes concolorus*	EU526394	[15]
DIP	*Drosophila littoralis*	FJ447340	UNP	MEG	*Sialis hamata*	FJ859905	[14]
DIP	*Drosophila mauritiana*	AF200830	[31]	RPH	*Mongoloraphidia harmandi*	FJ859902	[14]
DIP	*Drosophila melanogaster*	U37541	[32]	RPH	*Agulla *sp.	FJ207460-1	[13]
DIP	*Drosophila sechellia*	AF200832	[31]	MCP	*Neopanorpa pulchra*	FJ169955	UNP

ORD, order; N, identification number; ACN, accession number; REF, reference. COL, Coleoptera; DIP, Diptera; HYM, Hymenoptera; LEP, Lepidoptera, NEU, Neuroptera; MEG, Megaloptera; RPH, Raphidioptera; MCP, Mecoptera. UNP, unpublished mtDNA genome; **, **present paper**. Within each order taxa are listed alphabetically.

## Results and discussion

### Genome structure

**Figure 2:**
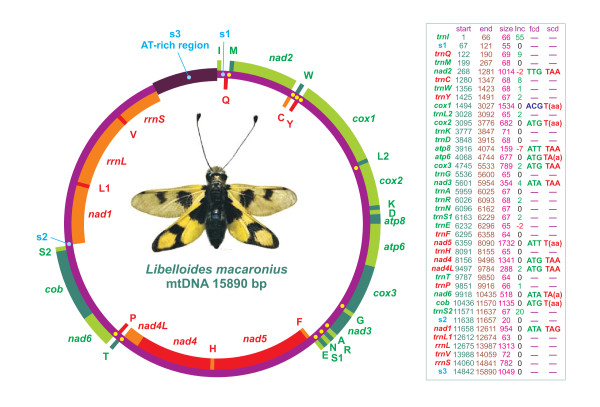
**Structure of the mitochondrial genome of *L. macaronius***. Genes are depicted as described in the legend of Figure 1. Intergenic spacers were marked with yellow/light-blue dots. Only spacers > 15 bp are labeled s1-s3. The genomic features are described in the table on the right. Start, first position along α strand; end, last position along α strand; size, size of the sequence; inc, intergenic nucleotides; fcd, first codon; scd, stop codon. Incomplete stop codons are written in parentheses. Negative inc values refer to overlapping nucleotides for genes located on the same or different strands.

The mtDNA genome of *L. macaronius *is a circular molecule 15,890 bp long (Figure 2). It contains the entire set of 37 genes usually present in animal mtDNAs and 14 non-coding portions. Three of these spanning at least 15 bp are labeled as intergenic spacers (s1-s3) in Figure 2.

*Atp8 *and *atp6 *are the only genes, located on the same strand, that overlap. The *atp8*-*atp6 *partial superimposition is a common feature of neuropterid mtDNAs and, in general, of animal mtDNAs [1,13,15]. Other genes located on the same strand (both α and β) are contiguous (e.g., *nad4L *and *nad4*) or separated by a few nucleotides (e.g., *nad3 *and *trnA*). Genes on opposite strands overlap (e.g., *nad2 *and *trnC*), are contiguous (e.g., *trnQ *and *trnM*), or are separated by some nucleotides (e.g., *trnT *and *trnP*) or intergenic spacers (e.g., *trnS2 *and *nad1*) (Figure 2). Similar patterns can be observed in other neuropterid mtDNAs. It must be noted that *nad4L *and *nad4 *are contiguous in *A. appendiculatus *and *L. macaronius *(both members of the family Ascalaphidae). Conversely, in all other sequenced neuropterid mtDNAs, *nad4L *and *nad4 *overlap by 7 bp [13,15].

The gene order of *L. macaronius *mtDNA differs from the insect ancestral gene order in the translocation of *trnC*, which is located upstream of *trnW *in contrast to its traditional location downstream of *trnW *(Figures 1, 2). This translocation is shared by and exclusive to all neuropteran mtDNAs that are fully or partly sequenced to date. Conversely, Megaloptera and Raphidioptera exhibit the typical insect ancestral gene order as shown in Figure 1[13,15]. This figure presents the distribution of various gene orders in holometabolous insects, mapped on the tree obtained by Wiegmann et al. [53], but alternative phylogenetic hypotheses exist ([e.g., [54]]).

According to Aspöck et al., Neuroptera includes three major groups, Hemerobiiformia, Myrmelontiformia and Nevrorthiformia [55]. The first two taxa are sister groups and encompass most neuropteran species, while Nevrorthiformia includes the single small family Nevrorthidae, which represents an early branching-off taxon within Neuroptera [55,56]. This view has been challenged by Winterton et al., who favor a paraphyletic Hemerobiiformia with respect to Myrmelontiformia and identifies the Coniopterygidae (Hemerobiiformia) family as the sister taxon of all other Neuroptera [57]. Irrespective to the arrangement of internal taxa, all studies strongly support a monophyletic Neuroptera order [55-57].

Available neuropteran mtDNAs, fully or partly sequenced, have been obtained from species belonging to distinct phyletic lines within Neuroptera. Indeed *D. latistyla *and *P. punctatus *belong to the divergent hemerobiiform families Mantispidae and Polystoechotidae, while *M. immaculatus *(Myrmelontidae), and *A. appendiculatus *+ *L. macaronius *(Ascalaphidae) are members of two myrmelontiform families [13,14], present paper]. The known distribution of *trnC *translocations was mapped (data not shown) based on the recent phylogeny of Neuropterida produced by Winterton et al., which also contains estimations of the divergence times of major phyletic lineages [57]. It appears that such genomic re-arrangement occurred at minimum 238 million years ago. The estimation could be further extended back to 294 million years if this genomic change is confirmed for the whole order Neuroptera.

Further work is needed to establish whether the *trnC *translocation represents a strong molecular signature for the entire Neuroptera clade.

Within Endopterygota, the translocation of *trnM *characterizes all lepidopteran species sequenced to date and seems to be a strong molecular mitochondrial signature for the whole order (Figure 1) (e.g., [11,12]). At the family level, the mosquitoes (family Culicidae) are characterized by the *trnR*-*trnA *arrangement instead of the traditional *trnA*-*trnR *(Figure 1) [9,10]. Several other re-arrangements are known for endopterygote mtDNAs but are restricted to single species (e.g., *Xenos vesparum*) or not consistent within broad taxonomic groups (e.g., Hymenoptera) [37,39,58-63]. The insect ancestral gene order remains the most widespread arrangement among holometabolous (Figure 1) and heterometabolous insects, suggesting that the gene order cannot play a pivotal role in resolving the relationships at the interordinal level among pterygote insects [7]. Conversely, mtDNA re-arrangements represent strong potential signatures to define taxa at the order and lower taxonomic levels (i.e., family, genus and lower) [9-14,39], present paper].

### Base composition and AT- and GC-skews in the mitochondrial genomes of endopterygote insects

**Figure 3:**
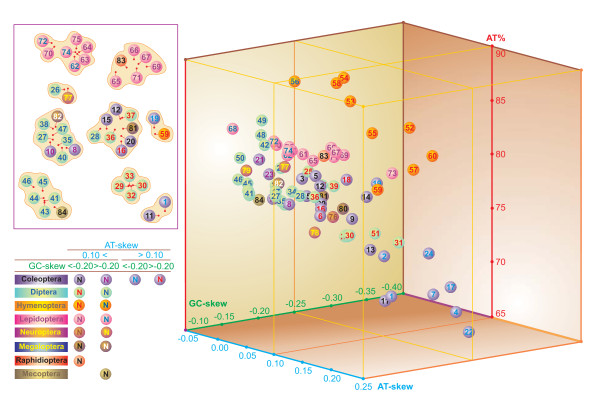
**Three-dimensional scatter-plot of the AT-, GC-skew and A+T% in endopterygote insects**. Values calculated on the α strand of full-length mtDNA genomes. Species with a placement that is difficult to identify in the main plot are depicted in the frame. Taxa are numbered as in Table 2.

The nucleotide-compositional behavior of mitochondrial genomes is usually investigated through the calculation of the three parameters: AT-skew, GC-skew, and A+T content (AT%), expressed in percentages (e.g., [8,12]). Here these parameters were studied for 84 complete endopterygotan mtDNAs (Table 2; Additional file 1, Table S1) and combined, for the first time, in a single three-dimensional scatter-plot analysis (Figure 3).

**Table 2:** AT%, AT-skew and GC-skew in endopterygotan complete mtDNAs

ORD	N	TAXON	AT%		AT-skew		GC-skew		ORD	N	TAXON	AT%		AT-skew		GC-skew	
COL	1	*Acmaeodera *sp.	A1	68.41	B4	0.1142	C4	-0.2492	DIP	43	*Drosophila sechellia*	A3	77.57	B2	0.0092	C6	-0.1351
COL	2	*Adelium *sp	A2	72.71	B4	0.1367	C4	-0.2222	DIP	44	*Drosophila simulans*	A3	77.93	B2	0.0084	C6	-0.1352
COL	3	*Anoplophora glabripennis*	A3	78.31	B2	0.0116	C4	-0.2066	DIP	45	*Drosophila yakuba*	A3	78.59	B2	0.0050	C6	-0.1364
COL	4	*Apatides fortis*	A1	67.19	B5	0.1704	C3	-0.2946	DIP	46	*Haematobia irritans*	A3	79.07	B2	0.0044	C6	-0.1245
COL	5	*Chaetosoma scaritides*	A3	79.04	B2	0.0427	C4	-0.2008	DIP	47	*Lucilia sericata*	A3	77.61	B2	0.0159	C5	-0.1666
COL	6	*Chauliognathus opacus*	A3	76.86	B4	0.1120	C5	-0.1532	DIP	48	*Mayetiola destructor*	A4	84.12	B3	0.0614	C6	-0.1100
COL	7	*Chrysochroa fulgidissima*	A1	69.92	B6	0.2026	C4	-0.2373	DIP	49	*Rhopalomyia pomum*	A5	85.15	B2	0.0468	C6	-0.1230
COL	8	*Crioceris duodecimpunctata*	A3	76.89	B2	0.0442	C5	-0.1634	DIP	50	*Simosyrphus grandicornis*	A4	80.84	B1	-0.0038	C6	-0.1328
COL	9	*Cyphon *sp.	A3	75.17	B3	0.0721	C4	-0.2282	DIP	51	*Trichophthalma punctata*	A2	73.96	B3	0.0912	C4	-0.2447
COL	10	*Hydroscapha granulum*	A3	77.29	B2	0.0297	C5	-0.1519	HYM	52	*Abispa ephippium*	A4	80.61	B1	-0.0186	C1	-0.3795
COL	11	*Lucanus mazama*	A1	67.11	B3	0.0743	C3	-0.2723	HYM	53	*Apis mellifera ligustica*	A4	84.86	B2	0.0183	C3	-0.2686
COL	12	*Macrogyrus oblongus*	A3	78.00	B2	0.0421	C4	-0.2080	HYM	54	*Bombus ignitus*	A5	86.78	B2	0.0028	C3	-0.2719
COL	13	*Mordella atrata*	A2	71.94	B3	0.0691	C3	-0.2556	HYM	55	*Cephus cinctus*	A4	81.95	B2	0.0343	C3	-0.2855
COL	14	*Priasilpha obscura*	A3	76.5	B3	0.0523	C3	-0.2646	HYM	56	*Diadegma semiclausum*	A5	87.41	B2	0.0086	C5	-0.1984
COL	15	*Psacothea hilaris*	A3	76.63	B2	0.0114	C4	-0.2129	HYM	57	*Evania appendigaster*	A3	77.77	B2	0.0267	C2	-0.3491
COL	16	*Pyrocoelia rufa*	A3	77.41	B4	0.1058	C5	-0.1584	HYM	58	*Melipona bicolor*	A5	86.72	B2	0.0155	C3	-0.2511
COL	17	*Pyrophorus divergens*	A1	69.44	B5	0.1650	C3	-0.2915	HYM	59	*Orussus occidentalis*	A3	76.21	B2	0.0169	C2	-0.3076
COL	18	*Rhagophthalmus lufengensis*	A3	79.63	B4	0.1038	C5	-0.1959	HYM	60	*Vanhornia eucnemidarum*	A4	80.11	B3	0.0857	C2	-0.3282
COL	19	*Rhagophthalmus ohbai*	A3	79.15	B4	0.1185	C4	-0.2256	LEP	61	*Acraea issoria*	A3	79.76	B1	-0.0234	C4	-0.2352
COL	20	*Rhopaea magnicornis*	A3	75.59	B2	0.0050	C4	-0.2380	LEP	62	*Adoxophyes honmai*	A4	80.39	B1	-0.0010	C5	-0.1964
COL	21	*Sphaerius *sp.	A4	80.68	B2	0.0104	C6	-0.1482	LEP	63	*Antheraea pernyi*	A4	80.16	B1	-0.0214	C4	-0.2163
COL	22	*Tetraphalerus bruchi*	A1	66.99	B6	0.2477	C3	-0.2515	LEP	64	*Antheraea yamamai*	A4	80.29	B1	-0.0221	C4	-0.2199
COL	23	*Trachypachus holmbergi*	A3	79.45	B2	0.0205	C5	-0.1536	LEP	65	*Artogeia melete*	A3	79.78	B2	0.0121	C4	-0.2218
COL	24	*Tribolium castaneum*	A2	71.68	B4	0.1091	C2	-0.3053	LEP	66	*Bombyx mandarina*	A4	81.68	B3	0.0547	C4	-0.2131
DIP	25	*Aedes aegypti*	A3	79.00	B2	0.0169	C4	-0.2109	LEP	67	*Bombyx mori*	A4	81.32	B3	0.0587	C4	-0.2162
DIP	26	*Aedes albopictus*	A3	79.54	B2	0.0079	C5	-0.1812	LEP	68	*Coreana raphaelis*	A4	82.66	B1	-0.0474	C5	-0.1578
DIP	27	*Anopheles gambiae*	A3	77.56	B2	0.0322	C5	-0.1540	LEP	69	*Diatraea saccharalis*	A4	80.02	B2	0.0213	C3	-0.2575
DIP	28	*Anopheles quadrimaculatus A*	A3	77.36	B2	0.0406	C5	-0.1814	LEP	70	*Eriogyna pyretorum*	A4	80.82	B1	-0.0305	C4	-0.2047
DIP	29	*Bactrocera carambolae*	A2	73.55	B3	0.0656	C4	-0.2237	LEP	71	*Lymantria dispar*	A3	79.88	B2	0.0160	C4	-0.2473
DIP	30	*Bactrocera dorsalis*	A2	73.58	B3	0.0675	C4	-0.2283	LEP	72	*Manduca sexta*	A4	81.79	B1	-0.0053	C5	-0.1811
DIP	31	*Bactrocera oleae*	A2	72.63	B3	0.0884	C3	-0.2802	LEP	73	*Ochrogaster lunifer*	A3	77.84	B2	0.0301	C2	-0.3175
DIP	32	*Bactrocera papayae*	A2	73.52	B3	0.0662	C4	-0.2263	LEP	74	*Phthonandria atrilineata*	A4	81.02	B2	0.0066	C5	-0.1921
DIP	33	*Bactrocera philippinensis*	A2	73.63	B3	0.0656	C4	-0.2244	LEP	75	*Saturnia boisduvalii*	A4	80.62	B1	-0.0240	C4	-0.2170
DIP	34	*Ceratitis capitata*	A3	77.48	B2	0.0211	C5	-0.1851	NEU	76	*Ascaloptynx appendiculatus*	A3	75.57	B3	0.0676	C4	-0.2059
DIP	35	*Chrysomya putoria*	A3	76.70	B2	0.0204	C5	-0.1701	NEU	77	*Ditaxis biseriata*	A3	79.79	B2	0.0154	C5	-0.1793
DIP	36	*Cochliomyia hominivorax*	A3	76.90	B2	0.0344	C4	-0.2067	NEU	78	*Libelloides macaronius*	A2	74.5	B3	0.0719	C5	-0.1767
DIP	37	*Culicoides arakawae*	A3	77.36	B2	0.0092	C4	-0.2369	NEU	79	*Polystoechotes punctatus*	A3	78.96	B1	-0.0287	C5	-0.1612
DIP	38	*Cydistomyia duplonotata*	A3	77.93	B2	0.0031	C5	-0.1771	MEG	80	*Corydalus cornutus*	A2	74.90	B2	0.0140	C3	-0.2620
DIP	39	*Dermatobia hominis*	A3	77.82	B2	0.0429	C4	-0.2267	MEG	81	*Protohermes concolorus*	A3	75.83	B1	-0.0111	C3	-0.2539
DIP	40	*Drosophila littoralis*	A3	76.24	B2	0.0124	C5	-0.1826	MEG	82	*Sialis hamata*	A3	78.32	B2	0.0145	C5	-0.1711
DIP	41	*Drosophila mauritiana*	A3	77.71	B2	0.0094	C6	-0.1348	RPH	83	*Mongoloraphidia harmandi*	A4	80.31	B2	0.0230	C4	-0.2277
DIP	42	*Drosophila melanogaster*	A4	82.16	B2	0.0167	C6	-0.1504	MCP	84	*Neopanorpa pulchra*	A3	76.38	B1	-0.0139	C5	-0.1676

ORD, order; N, identification number in Figure 3; COL, Coleoptera; DIP, Diptera; HYM, Hymenoptera; LEP, Lepidoptera, NEU, Neuroptera; MEG, Megaloptera; RPH, Raphidioptera; MCP, Mecoptera. AT%, A+T content expressed in percent. A1 (65.00 < AT% < 70.00); A2 (70.00 < AT% < 75.00); A3 (75.00 < AT% < 80.00); A4 (80.00 < AT% < 85.00); A5 (85.00 < AT% < 90.00). B1 (-0.050 < AT-skew ≤ 0.000); B2 (0.000 < AT-skew ≤ 0.050); B3 (0.050 < AT-skew ≤ 0.100); B4 (0.100 < AT-skew ≤ 0.150); B5 (0.150 < AT-skew ≤ 0.200) B6 (0.200 < AT-skew ≤ 0.250). C1 (-0.400 < GC-skew < -0.350); C2 (-0.300 < GC-skew < -0.250); C3 (-0.300 < GC-skew < -0.250); C4 (-0.250 < GC-skew < -0.200); C5 (-0.200 < GC-skew < -0.150); C6 (-0.150 ≤ GC-skew < -0.100). Within each order taxa are listed alphabetically.

Arbitrary partitioning schemes, further detailed below, were applied to the estimated values to facilitate the presentation/discussion of the results. Each empirical clustering scheme was defined in order to maximize the appreciation of the uneven distribution of various mtDNAs within the continuum of variation of the considered parameter.

#### A+T content

The range of variation of AT% spanned from 66.99 to 87.41, with the average value equal to 77.77. Different mtDNAs exhibited an uneven distribution within this range. Five principal clusters (A1-A5) were obtained that divided the whole range of AT% into intervals encompassing 5% of the variation.

The majority (61/84) of endopterygote mtDNAs exhibited an AT% in the range of 74-82 (A2 partially; A3; A4 partially), with a strong concentration in the interval 75-80 (43/84) (A3). Most of neuropterids were included in cluster A3, while *L. macaronius *and *C. cornutus *belonged to cluster A2. The mtDNA of *M. harmandi *was comprised in cluster A4. Very high AT% (>84) contents (A4 partially; A5) were characteristic of and limited to a few hymenopterans and dipterans. Conversely, low AT%s (< 70) were restricted to coleopterans (A1).

#### AT-skews

The average AT-skew was 0.039, while the whole range of variation extended from -0.047 to 0.248. The endopterygote mtDNAs were grouped into six clusters (B1-B6) through the partitioning of the whole range of AT-skews in intervals spanning 0.050.

The majority of mtDNAs exhibited a positive AT-skew (71/84) with two peaks, in the intervals 0.000-0.050 (44/84; B2) and 0.050-0.100 (16/84; B3). In cluster B2 were placed the mtDNAs of *C. cornutus, S. hamata, D. biseriata *and *M. harmandi*. The cluster B3 contained also the mtDNAs of *A. appendiculatus *and *L. macaronius*. Only some coleopteran mtDNAs exceed values of 0.100 (B4-B6), and the skews were higher than 0.200 solely in *C. fulgidissima *and *T. bruchi; (*B6). Negative AT-skews (13/84) were modest (>-0.005; B1), and lepidopteran taxa predominated in this group. The mtDNAs of *P. punctatus *and *P. concolorus *exhibited negative AT-skews.

#### GC-skews

The average GC-skew value was -0.213, and all 84 mtDNAs displayed negative skews. The whole set of genomes was grouped into six clusters (C1-C6), each spanning 0.050 of the range of GC-skew variation.

The majority of the GC-skews were located within the range -0.30/-0.15 (68/84; C3-C5), with two peaks represented by clusters C4 (30 genomes) and C5 (24 mtDNA). The neuropterids *C. cornutus *and *P. concolorus *were included in C3, *M. harmandi *and *A. appendiculatus in *C4, while *D. biseriata, L. macaronius, S. hamata*, and *P. punctatus *were inserted in cluster C5.

#### Overall comparison

When the three variables analyzed above were considered together, some patterns emerged. Even if the parameter distribution followed a continuum range of variation, most of the sequenced endopterygote mtDNAs exhibited A+T contents in the range 70%-80%, with the highest density in the interval 75%-80%; AT-skews in the range 0.003-0.010; and GC-skews in the interval -0.300-0.150. This behavior can be represented by the cluster formula: A2/A3,B2/B3,C3-C5. Most neuropterid mtDNAs are included in this formula, with the exceptions of *P. punctatus *and *P. concolorus *(B1).

While no genome exhibited extreme values for any of the three parameters, some mtDNAs markedly departed from the most represented ranges for the behavior of one or two parameters. Very low AT content was coupled with high AT-skew in some coleopteran mtDNAs. Very high A+T contents (>84%) were restricted to a small group of hymenopterans and dipterans. Highly negative GC-skews (< -0.300) were found mostly in hymenopterans, plus the moth *O. lunifer *and the beetle *T. castaneum*.

The comparisons performed at the intra-ordinal level showed that in several cases the extremes of the ranges of variation of the analyzed parameters were occupied by the same mtDNAs. This behavior could be the result of limited taxon sampling. Thus, further data are necessary to verify if the observed patterns are consistent over a broader taxon sampling.

Generally, the A+T content, AT-skew, and GC-skew exhibited complex behaviors that do not appear to be tightly linked, at least at the order level.

### Start/stop codons in neuropterid PCGs

The inferred start/stop codons for neuropterid PCGs are listed in Table 3. ATN, GTG, TTG, and GTT are accepted canonical mitochondrial start codons for invertebrate mtDNs and most of PCGs exhibited these start codons [64].

**Table 3:** Start/stop codons in neuropterid mitochondrial genes

Taxon	***nad2***		***cox1***		***cox2***		***atp8***		***atp6***		***cox3***		***nad3***	
	**ST**	**EN**	**ST**	**EN**	**ST**	**EN**	**ST**	**EN**	**ST**	**EN**	**ST**	**EN**	**ST**	**EN**

*Ascaloptynx appendiculatus*	ATT	TAA	TTA	Taa	ATG	Taa	ATT	TAA	ATG	TAa	ATG	TAA	ATA	TAA
*Ditaxis biseriata*	ATT	TAA	ATT	Taa	ATG	Taa	ATT	TAA	ATG	TAa	ATG	TAA	ATT	TAA
*Libelloides macaronius*	TTG	TAA	ACG	Taa	ATG	Taa	ATT	TAA	ATG	TAa	ATG	TAA	ATA	TAA
*Myrmelon immaculatus*	ATA	TAA	ACG	Taa	ATG	Taa	ATT	TAA	ATG	TAG	ATG	n.a.	n.a	n.a
*Polystoechotes punctatus*	ATT	TAA	TCG	Taa	ATG	Taa	ATT	TAA	ATG	TAA	ATG	TAa	ATT	TAA
*Corydalus cornutus*	ATT	TAA	ATT	TAa	ATG	Taa	ATT	TAA	ATG	TAa	ATG	Taa	ATT	Taa
*Protohermes concolorus*	ATT	TAa	ATT	TAA	ATG	TAA	ATT	TAA	ATG	TAa	ATG	Taa	ATT	Taa
*Sialis hamata*	ATT	TAA	ATT	Taa	ATG	Taa	ATC	TAA	ATG	TAa	ATG	TAA	ATT	TAA
*Agulla *sp.	n.a.	n.a.	CTG	Taa	ATT	n.a.	n.a.	n.a.	n.a.	n.a.	n.a.	n.a.	n.a.	n.a.
*Mongoloraphidia harmandi*	ATT	TAa	CTG	Taa	ATA	Taa	ATC	Taa	ATG	TAa	ATG	TAa	ATC	Taa

**Taxon**	***nad5***		***nad4***		***nad4L***		***nad6***		***cob***		***nad1***			

	ST	EN	ST	EN	ST	EN	ST	EN	ST	EN	ST	EN		

*Ascaloptynx appendiculatus*	ATA	Taa	ATG	TAa	ATG	TAA	ATC	TAA	ATG	Taa	ATA	TAG		
*Ditaxis biseriata*	ATT	Taa	ATG	Taa	ATG	TAA	ATT	TAA	ATG	Taa	TTG	TAA		
*Myrmelon immaculatus*	n.a.	n.a.	n.a.	n.a.	n.a.	n.a.	n.a.	n.a.	n.a.	Taa	n.a.	TAG		
*Libelloides macaronius*	ATT	Taa	ATG	TAA	ATG	TAA	ATA	TAa	ATG	Taa	ATA	TAG		
*Polystoechotes punctatus*	ATT	Taa	ATG	Taa	ATG	TAA	ATC	TAa	ATG	Taa	TTG	TAG		
*Corydalus cornutus*	ATT	Taa	ATG	Taa	ATG	TAA	ATT	TAa	ATG	Taa	TTG	TAA		
*Protohermes concolorus*	ATT	Taa	ATG	Taa	ATG	TAA	ATT	TAa	ATG	Taa	TTG	TAA		
*Sialis hamata*	ATT	Taa	ATG	Taa	ATG	TAA	ATT	TAa	ATG	Taa	ATG	TAA		
*Agulla sp.*	n.a.	n.a.	n.a.	n.a.	n.a.	n.a.	n.a.	n.a.	n.a.	TAA	n.a.	TAA		
*Mongoloraphidia harmandi*	ATA	TAA	ATG	Taa	ATT	TAA	ATA	TAa	ATG	TAA	TTG	TAA		

ST, start codon; EN, end (stop) codon; TAa and Taa, stop codon incomplete; n.a., not available.

The *cox1 *start codon for *L. macaronius *was inferred to be the triplet ACG, a non-canonical start codon for mtDNA genes. Despite the fact that *cox1 *is one of more conserved mitochondrial genes, the identification of its start codon has been problematic (see [13]). An ATC codon located 27 bp upstream of ACG, within the *trnY *gene that is encoded on the β strand, could be a start codon for *L. macaronius cox1*. However, amino acid sequence comparison with a broad selection of endopterygote insects led us to the more probable conclusion that the ACG triplet was the start codon for *L. macaronius cox1*. An ACG start codon for *cox1 *has also been proposed for *M. immaculatus *[13]. The non-canonical start codons TTA and TCG have previously been inferred for *cox1 *in *A. appendiculatus *and *P. punctatus *[13]. The remaining neuropterid *cox1 *genes exhibited an ATT start codon. Non-canonical start codons in *cox1 *have been inferred repeatedly for endopterygote insects (e.g., [12,65,66]).

Stop codons for the 13 neuropterid PCGs were almost invariably complete (TAA) or incomplete TAa/Taa. The only exception was observed in *nad1*, where the two ascalaphids *L. macaronius *and *A. appendiculatus *and *P. punctatus *exhibited TAG as stop codon.

### Codon usage and amino acids abundance

**Figure 4:**
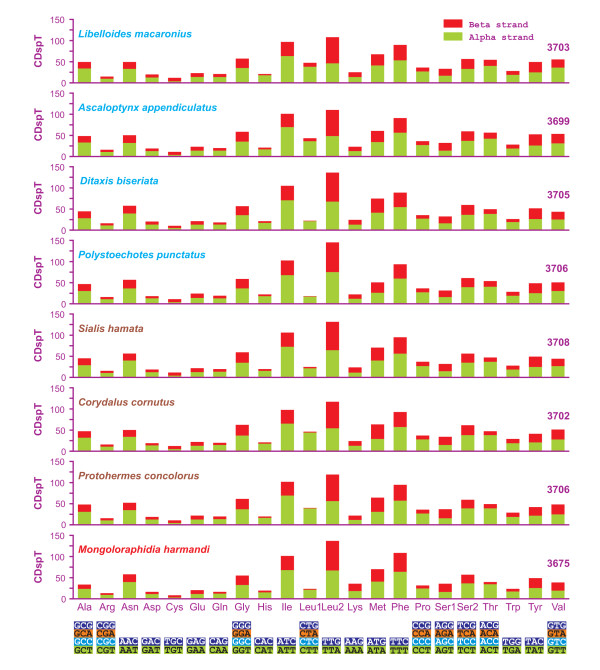
**Codon distribution in neuropterid mtDNAs**. Numbers to the left refer to the total number of codons. CDspT, codons per thousand codons. Codon families are provided on the *x*-axis. Within each family, the percentage of codons located, respectively, on the α strand or β strand is colored blue or red.

**Figure 5:**
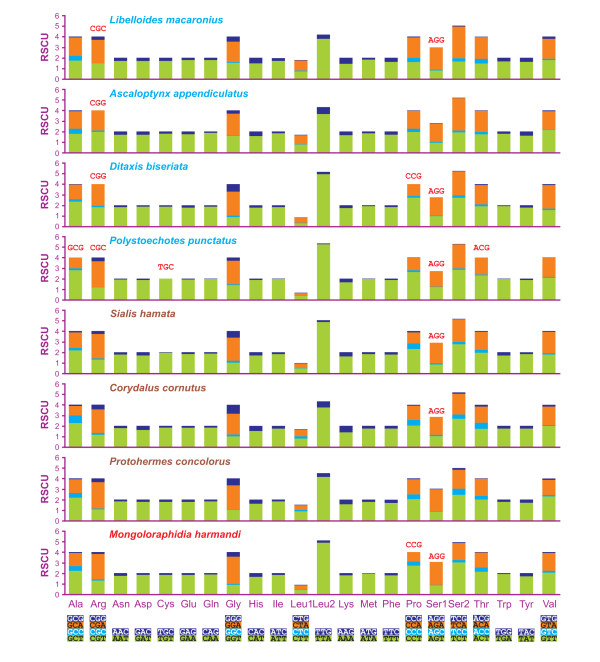
**Relative synonymous codon usage (RSCU) in neuropterid mtDNAs**. Codon families are provided on the *x*-axis. Red codon, codon not present in the genome.

All analyzed neuropterid mtDNAs use the standard invertebrate mitochondrial genetic code. This result was determined using the server GenDecoder [67,68]. The behavior of codon families in PCGs was investigated and the results are presented in Figures 4, 5. First codons, as well as stop codons, complete and incomplete, were excluded from the analysis to avoid biases due to unusual putative start codons and incomplete stop codons. The abundance of codon families and relative synonymous codon usage (RSCU) were calculated together with the other statistics listed below.

The A+T content, AT-skew, G+C content and GC-skew, which were calculated in pooled-α + β, pooled-α and pooled-β PCGs, exhibited behaviors identical, similar to or contrasting with those observed for the whole genomes (see Additional file 2, Table S2).

The total number of codons was different in analyzed species (Figure 4; Additional file 2, Table S2). The codon families exhibiting more than 50 codons per thousand cds varied (7-9) and the majority of them were two-fold degenerate in codon usage and AT-rich. In almost all mtDNAs, the four most represented codon families were Leu2, Ile, Phe and Met with Leu2 being the most common. The only exception was *P. punctatus*, in which Ser2 was the fourth most abundant codon family, while Met family occupied only the seventh position. Leu, Ile, Ser and Phe were the most abundant amino acids, and their sum varied from 42.75% (*L. macaronius*) to 46.62% (*M. harmandi*) of the total number of amino acids.

Four-fold degenerate codon usage was A/T biased in the third position, and T was the preferred nucleotide, except in the Arg, Gly and Ser1 codon families, where A was the most common nucleotide in the third position. Two-fold degenerate codon usage showed a similarly biased pattern, with A/T favored over G/C in the third position (Figure 5). Both patterns were in agreement with the AT-biased content exhibited by pooled-α+β PCGs.

In pooled-α PCGs, the four-fold degenerate codon usage followed a pattern similar to that of the whole PCG set, with the exceptions of Ala, Pro and Val codon families (Figure 5 vs. Additional file 3, Figure S1).

In the pooled-β PCGs, the four-fold degenerate codon usage partly mirrored the pattern observed for pooled-α+β PCGs. Among the discrepancies, the loss of synonymous GC-rich codons was the easiest to detect (Figure 5 vs. Additional file 4, Figure S2).

The behavior of pooled-α+β PCGs in different species varied with respect to the usage of the whole set (62) of codons encoding amino acids. All missed codons were GC-rich. Codon usage could not be directly linked to total number of codons nor to A+T content (Figure 5; Additional file 2, Table S2). The reduction in codon usage could be possibly linked to AT/GC-skews. Indeed, *P. punctatus*, which used the smallest number of codons, also exhibited the most negative AT-skew_α+β _and the highest GC-skew_α+__β_. The number of used codons decreased when PCGs encoded on a single strand were analyzed (Additional file 2, Table S2; Additional file 3, Figure S1; Additional file 4, Figure S2). The pattern observed for pooled-α+β PCGs was mostly mirrored by pooled-α PCGs. On the β strand, codon reductions appeared to be species specific. The lost codons belonged to GC-rich and comparatively A-rich codon-families. Finally, on the β strand, the pattern of codon loss appeared to be linked to the single/combined effects of GC content and AT-/GC-skews.

The total number of α-codons vs. the total number of β-codons (ratio_α/β_) was calculated for every species (Table 4). The overall (o) average (avg) ratio_α/β _(1.59 ± 4 × 10^-3^) was used as a measure to identify possible distributional biases of different codon families. Several codon families exhibited ratio_α/β _values close to oavg-ratio_α/β_, suggesting that their distribution is not markedly diverse in the PCGs coded on different strands. Codon families His, Leu1, Pro, and Thr exhibited ratio_α/β _values strongly biased toward the α strand. Conversely, codon families Cys, Leu2 and Ser1 presented ratio_α/β _values clearly β strand-oriented.

**Table 4:** Total number of α-codons vs. total number of β-codons ratio_α/β_

codon family	**avg-ratio**_**α/β**_	STD
oavg	1.59	0.004
Ala	1.94	0.14
Arg	1.81	0.08
Asn	2.01	0.18
Asp	2.03	0.28
Cys	0.56	0.08
Glu	1.40	0.09
Gln	2.22	0.21
Gly	1.50	0.05
His	4.41	0.25
Ile	2.06	0.11
Leu1	13.22	7.46
Leu2	0.89	0.10
Lys	1.10	0.12
Met	1.18	0.24
Phe	1.54	0.12
Pro	3.01	0.22
Ser1	0.83	0.10
Ser2	1.66	0.17
Thr	3.67	1.06
Trp	2.18	0.28
Tyr	1.02	0.03
Val	1.37	0.30

Oavg, overall (o) average(avg) ratio_α/β_; avg-ratio_α/β_, average-ratio_α/β_; STD, standard deviation

The most obvious bias was due to structural/functional requirements of PCGs. This point is well represented by the distribution of Cys codon family. Cys was the rarest amino acid in mtDNA proteins and was more abundant in polypeptides (NADH subunits) encoded on the β strand than in the proteins encoded on the α strand. The Cys residues can produce intra- and inter-chain disulfide bridges (S-S) that play a fundamental structural role. Disulfide bonds and interactions between pairs of Cys residues have been postulated to have an important structural role in NADH subunits of primate mtDNA [69]. The abundance of Cys residues in neuropterid NADHs, and in general in endopterygotes, appears to agree with findings derived from primates. Several marked departures from oavg-ratio_α/β _can be explained in terms of structural/functional requirements of single polypeptides and are not explored here.

A compositional bias effect was evident in the behavior of Leu1 (CTN) and Leu2 (TTR) codon families. As mentioned above, Leu was the most abundant amino acid in the PCGs. The pooled superfamily Leu = Leu1 + Leu2 exhibited an avg-ratio_α/β _= 1.28 ± 0.11. This result supports a moderate β strand bias in the distribution of Leu. However, Leu1 and Leu2 codon families exhibited an opposite bias in term of strand distribution. These strand biases can be linked to the AT-skew and GC content shown by pooled-α and pooled-β PCGs.

### Behavior of PCGs in neuropterid mtDNAs

Different parameters have been used to evaluate the performance of mitochondrial single/combined PCGs for phylogenetic, population genetics, and taxon molecular identification purposes (e.g., [11,12]). The suitability of different PCGs is linked to, influenced by the evolutionary history of each molecular marker. The calculation of p-distance values has been used to test the level of divergence within each PCG (e.g., [11]). Recently, codon usage has also been investigated to study the differentiation of mitochondrial PCGs [12]. In the present paper, the evolutionary behavior of PCGs, at both the nucleotide and amino acid levels, was studied in a combined analysis based on the calculation/estimation of p-distances, effective number of codon usage (ENC), phylogenetic signal and the saturation of the substitution process present in single/combined PCGs.

The percent of fully resolved quartets (%FRQ), obtained in a maximum likelihood mapping analysis, was used as a measure of phylogenetic signal [70]. The saturation of the substitution process was established by calculating the m-slope of the regression line passing through the origin in a scatter plot analysis of p-distances vs. GTR+I+G - mtREV24/JTT + G distances (see Methods for further details).

**Figure 6:**
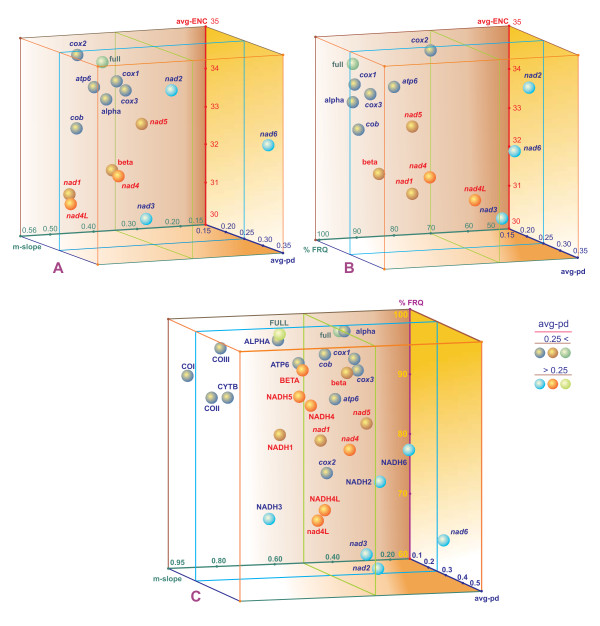
**Three-dimensional scatter plot graphics calculated for PCGs**. Dots correspond to average values calculated for different genes/proteins. PCGs/proteins on the α strand are blue, PCGs/proteins on the β strand are red. Pooled-α + β PCGs/proteins are green. Gene/protein nomenclature is as in main text and Table 5. A) ENC vs. avg-p-distance vs. m-slope; B) ENC vs. avg-p-distance vs. % of fully resolved quartets; C) % of fully resolved quartets vs. m-slope vs. avg-p-distance.

Parameters were estimated for pooled-α+β, pooled-α, pooled-β, and single PCGs at both the nucleotide and amino acid levels, with the exception of *atp8*. This gene was not analyzed individually because it contains a limited number of codons (< 55) to provide reliable estimations of ENC [71]. Conversely, *atp8 *was included in the pooled-α+β/-α sets. The results of the global analysis are summarized in Figure 6 and Table 5.

**Table 5:** Phylogenetic signal, saturation of substitution process, p-distances, and effective codon usage in neuropterid PCGs

pcg/PROTEIN	%FRQ	m-slope	avg-pd	avg-ENC
pooled-(α+β) (full)	100	0.466	0.247 ± 0.034	34.758 ± 2.640
POOLED-(α+β) (FULL)	100	0.684	0.262 ± 0.062	n.a.c.
pooled-α (alpha)	100	0.458	0.248 ± 0.030	33.783 ± 2.111
POOLED-α (ALPHA)	98.6	0.676	0.238 ± 0.055	n.a.c.
pooled-β (beta)	92.86	0.442	0.246 ± 0.040	31.898 ± 1.603
POOLED-β (BETA)	94.3	0.625	0.297 ± 0.073	n.a.c.
*nad2*	61.43	0.392	0.346 ± 0.037	34.321 ± 3.279
NADH2	77.1	0.436	0.430 ± 0.067	n.a.c.
*cox1*	94.28	0.384	0.189 ± 0.023	33.991 ± 1.584
COI	91.4	0.908	0.105 ± 0.035	n.a.c.
*cox2*	75.71	0.491	0.209 ± 0.036	34.840 ± 4.054
COII	88.6	0.867	0.174 ± 0.076	n.a.c.
*atp6*	88.57	0.482	0.244 ± 0.034	34.105 ±2.770
ATP6	94.3	0.591	0.215 ± 0.061	n.a.c.
*cox3*	98.86	0.390	0.220 ± 0.032	33.890 ± 2.895
COIII	97.1	0.845	0.191 ± 0.059	n.a.c.
*nad3*	62.86	0.394	0.280 ± 0.032	30.720 ± 4.398
NADH3	70	0.751	0.313 ± 0.068	n.a.c.
*nad5*	84.28	0.377	0.249 ± 0.042	33.086 ± 2.306
NADH5	89.9	0.639	0.300 ± 0.078	n.a.c.
*nad4*	80.00	0.436	0.254 ± 0.044	31.771 ± 2.270
NADH4	88.6	0.608	0.315 ± 0.075	n.a.c.
*nad4L*	68.57	0.551	0.262 ± 0.044	31.150 ± 4.875
NADH4L	71.4	0.571	0.336 ± 0.067	n.a.c.
*nad6*	65.71	0.171	0.349 ± 0.045	32.722 ± 3.411
NADH6	82.8	0.360	0.468 ± 0.090	n.a.c.
*cob*	95.71	0.498	0.214 ± 0.030	32.932 ± 2.162
CYTB	88.6	0.639	0.182 ± 0.059	n.a.c.
*nad1*	81.43	0.516	0.217 ± 0.038	31.225 ± 2.100
NADH1	82.9	0.673	0.246 ± 0.072	n.a.c.

Pcg-PROTEIN, protein coding gene/relative protein; %FRQ, percent of fully resolved quartets obtained in a maximum likelihood mapping analysis [70]; m-slope, slope of the regression line passing through the origin in a scatter plot analysis of p-distances vs. GTR+I+G - mtREV24/JTT + G + G distances distances; avg-pd, average p-distance; avg-ENC, average-ENC (effective number of codon usage); n.a., not applicable calculation.

As a general rule, proteins, individually or pooled, exhibited levels of saturation of the substitution process lower than those observed in the nucleotidic counterparts. This result can be explained in terms of the substitution process, which permits more alternatives in the amino acid replacements. The m-slope values show that all PCGs/proteins experienced some degree of saturation of the substitution process. This phenomenon was particularly marked at the third codon positions of some PCGs, where the GTR+I+G-distance values largely exceeded 1 (data not shown), a result strongly supporting a complete saturation of the substitution process [72].

Pooled-α+β PCGs/proteins and pooled-α PCGs/proteins showed the best phylogenetic signals and clearly surpassed pooled-β PCGs/proteins. Because pooled-α+β and pooled-α proteins also exhibited a less pronounced saturation of the substitution process than their nucleotide counterparts, they represented the first choice as markers to investigate the phylogenetic relationships among Neuropterida at the inter-order taxonomic level.

The single genes exhibiting the best phylogenetic signals at the inter-order taxonomic level were *cob, cox1 *and *cox3 *(%FRQ> 94.00). Intermediate values were observed in *atp6, cox2, nad4, nad5 *and *nad1 *(89.00 < %FRQ > 75.00) The lowest signals were present in *nad2, nad3, nad4L *and *nad6 *(%FRQ < 70.00).

Pooled and single PCGs having the best phylogenetic signals usually exhibited a combination of high avg-ENCs, small avg-pds, and high m-slopes in their protein counterparts (e.g., *cox1*), but exceptions occurred. Conversely, PCGs with low phylogenetic signals usually showed high avg-pds and low values of m-slope at both the nucleotide and amino acid levels (e.g., *nad2*).

Reduction of phylogenetic signal with respect to the best-performing PCGs can be explained in terms of three different processes/properties: a) accelerated evolution; b) retarded evolution; and c) possession of an intrinsically lower phylogenetic signal.

A marked accelerated evolution mechanism can be invoked for *nad2, nad3 *and *nad6*. These PCGs showed avg-pds, at both the nucleotide and amino acid levels, which were clearly higher than those observed in *cox1, cox3 *and *cob*. Furthermore, their m-slope values favored the occurrence of a more pronounced saturation of the substitution process, a finding corroborated by the lowest values of fully resolved quartets. All of these results are compatible only with an acceleration of the substitution process. Thus, *nad2, nad3 *and *nad6 *should be excellent markers for studies performed at medium/very low taxonomic levels (i.e., family, genus, species).

The mechanism of accelerated evolution, but less pronounced than that described above, can be invoked also for *atp6, nad5 *and *nad4*. These genes showed avg-pds higher than *cox1, cox3 *and *cob*, at both the nucleotide and amino acid levels. They exhibited phylogenetic signals higher than *nad2, nad3 *and *nad6 *but lower than the best-performing PCGs. Finally, their m-slope values were comparable to those of *cox1, cox3 *and *cob*. All these findings suggest that *atp6, nad5 *and *nad4 *evolved slightly faster than the best-performing PCGs and that these genes can be optimally used at the order or lower taxonomic levels.

The *nad1 *and *cox2 *genes had avg-pds similar to or even smaller than *cox1, cox3 *and *cob*, while their levels of saturation of the substitution process appeared to be less marked than PCGs exhibiting the highest %FRQ values. These results suggest that the reduction of phylogenetic signal in *cox2 *and *nad1 *was probably due to a slower rate of divergence of these two genes in comparison with *cox1, cox3 *and *cob*. Thus, they should be considered favored markers at high/very high taxonomic ranks.

The behavior of *nad4L *provided contradictory evidence. The low %FRQ and relatively high avg-pd value suggested that this is a fast evolving gene and that the loss of phylogenetic signal was due to an accelerated rate of multiple substitutions. However, the m-value was the highest obtained in the analysis and did not favor a strong saturation of the substitution process in the evolution of this gene. Thus, it could be that even evolving in a way not dissimilar to *cox1, cox3 *and *cob, nad4L *has an intrinsically lower phylogenetic signal due to its reduced size.

Taken together, these data indicate that the 13 PCGs encoded by mtDNA exhibit complex evolutionary trajectories that can be investigated using the combination of the parameters considered above. No single parameter seems able to fully describe the behavior of PCGs.

### Comparison of tRNA structure in neuropterid genomes

**Figure 7:**
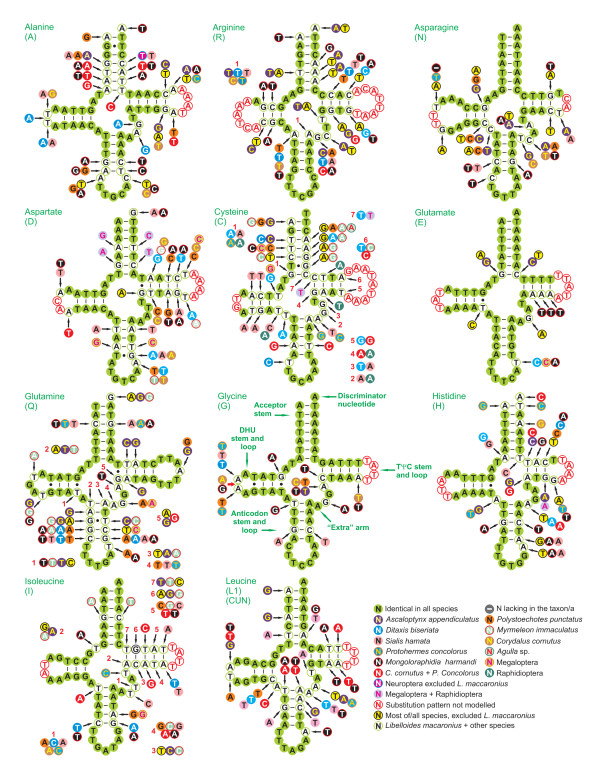
**Secondary structure of tRNA families (*trnA*-*trnL1*) in neuropterid mtDNAs**. The nucleotide substitution pattern for each tRNA family was modeled using as reference the structure determined for *L. macaronius*.

**Figure 8:**
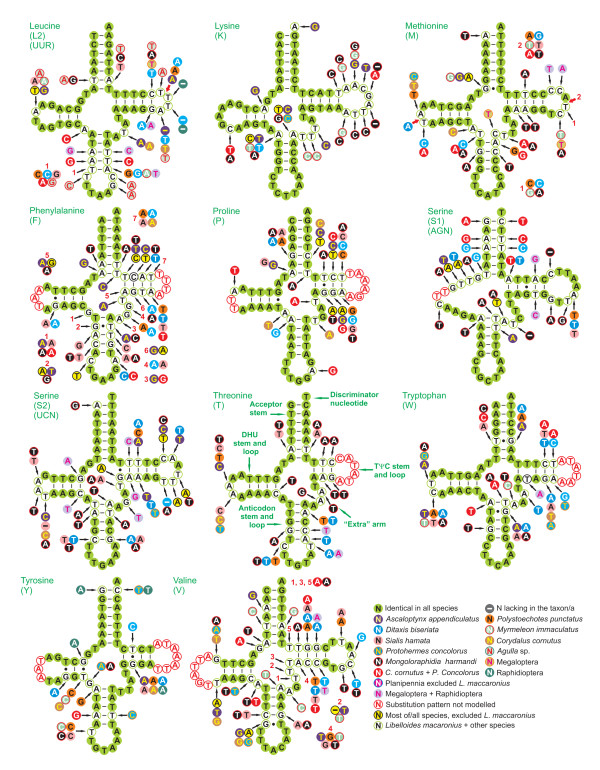
**Secondary structure of tRNA families (*trnL2*-*trnV*) in neuropterid mtDNAs**. The nucleotide substitution pattern for each tRNA family was modeled using as reference the structure determined for *L. macaronius*.

The mtDNA of *L. macaronius *contained all 22 tRNAs genes found in other neuropterid mtDNAs and typical of animal mitochondrial genomes [1]. All neuropterid mtDNAs had 14 α-strand tRNAs and 8 β-strand tRNAs (Figures 1, 2). Among the 22 tRNAs, only *trnS1 *did not exhibit the common cloverleaf structure, due to the absence of DHU stem. Loss of this stem in *trnS1 *is a feature common to many insect mtDNAs (e.g., [12]). The results of comparative analyses on secondary structures of neuropterid tRNAs are provided in Figures 7, 8. Multiple alignments of tRNAs genes were produced, and the percent of identical nucleotides (%INUC) was calculated for each group of orthologous sequences (Additional file 5, Table S3).

The pattern of nucleotide conservation was markedly α strand-biased. Indeed, *trnG, trnK, trnN, trnM *and *trnE*, which showed the highest levels of nucleotide conservation (%INUC ≥ 70), were all located on the α strand.

*TrnL2, trnP, trnQ, trnS1, trnS2, trnT, trnW *and *trnY *showed 60 < %INUC < 70. Only three of them were located on the β strand (Figure 2). Seven remaining tRNAs exhibited 50% < %INUC < 60. Four of them were located on the α strand, i.e., *trnI, trnA, trnD*, and *trnR*, while three were on the β strand, i.e., *trnL1, trnH *and *trnV*. %INUC values < 50% characterized the β-strand genes *trnF *and *trnC*. While the level of conservation was positively α strand-biased, no clear pattern could be identified for single tRNAs located at various points along the genome. For example, *trnK*, one of the most conserved tRNAs, was located immediately upstream of *trnD *(Figure 2), a much less conserved gene (see above).

The tRNAs closest to the control region (i.e., *trnV *and *trnI*), which is one of the start points of mtDNA replication, were not among most/less conserved. The same applied to *trnS2*, immediately upstream of the s2 spacer, where another center of mtDNA replication was located (see below). The abundance of codon families did not appear to be linked to the level of tRNA conservation. Indeed, none of the most abundant codon families (Leu2, Ile and Phe) exhibited the highest %INUCs.

Irrespective of the level of conservation, some tRNAs presented mismatched pairs in stems (e.g., A-A in the anticodon stem of *trnK*; T-T in the acceptor stem of *trnM*). These mismatches are common in arthropod mtDNAs and can be corrected through editing processes (e.g., [73]) or may represent unusual pairings [74]. Further research at the transcript level is necessary to better characterize this feature in neuropterid mtDNAs.

In the most conserved tRNAs the nucleotide substitutions are largely restricted to TΨC and DHU loops and extra arms (Figures 7, 8), with changes on acceptor and anticodon stems reduced to 0-3 fully compensatory base changes (cbcs) (e.g., G-C vs. A-T in the anticodon stem of *trnG*) and/or hemi-cbc (e.g., G-T vs. A-T on the acceptor stem of *trnM*) [75]. Note that in *trnG*, the most conserved tRNA, it was not possible to model the substitution pattern in the TΨC loop due to a high level of variation among orthologous sequences (Figure 7). With the increased variation, the number of cbcs and hemi-cbcs increased in stems, usually with a more marked substitution process in the TΨC stem (e.g., *trnI, trnV*). In several tRNAs it was not possible to model properly the substitution patterns in TΨC and DHU loops due to the high level of divergence among sequences. Cbcs and hemi-cbcs were restricted to a single species or characterized taxa at a higher taxonomic rank (family/order). An example of the first type is the G-C pair found in the *trnH *acceptor harm of *P. concolorus*, which was mirrored by A-T in all other neuropterids (Figure 7). An example of a full cbc characterizing a unique family is the G-C pair found in the acceptor stem of *trnEs *of family Ascalaphidae (*A. appendiculatus *and *L. macaronius*), while other taxa exhibited the A-T pair (Figure 7). A substitution pattern involving two full cbcs characterizing the *trnS1 *acceptor arm of *C. cornutus *and *P. concolorus *(Corydalidae megalopterans) is another example of high-taxonomic rank cbcs (Figure 8). Figures 7, 8 depict several more examples.

The presence of hemi-cbcs and, particularly, full cbcs characterizing taxa at different taxonomic levels underscores the potential phylogenetic value of tRNA sequences, especially when secondary structures are taken into account. Until now, tRNAs have been rarely considered in the study of insect phylogenetic relationships [76,77]. Our results suggest that these markers deserve much more attention and should be more routinely used, as demonstrated by analyses performed in other animal groups (e.g., [78]).

**Figure 9:**
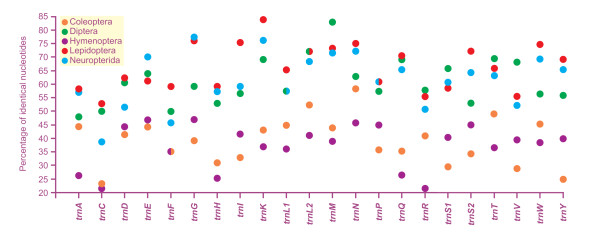
**Conservation of tRNA families in Endopterygota mtDNAs**. The percentage of identical nucleotides for each tRNA family was inferred from a multiple alignment produced with ClustalW [90] and refined manually, taking into account the secondary structure.

The study of the level of conservation in tRNA families was extended to other endopterygotan orders in which at least two fully sequenced mtDNAs exist. The results of this analysis are summarized in Figure 9.

The pattern of %INUC variation exhibited by codon families was not consistent among different endopterygotan taxa. The highest level of conservation was observed in lepidopteran *trnK *(%INUC = 84.51), while the minimum was found in hymenopteran *trnC *(%INUC = 20.55). Lepidoptera had nine codon families with %INUC ≥ 70, whereas five such families were found in Neuropterida, two in Diptera, and none in Coleoptera or Hymenoptera. Lepidopteran codon families were the most conserved (avg-%INUC = 66.25 ± 8.49), immediately followed by neuropterid codon families (avg-%INUC = 61.46 ± 9.84). Dipteran codon families showed slightly more divergence (avg-%INUC = 60.84 ± 8.59), while coleopteran (avg-%INUC = 38.92 ± 8.84) and hymenopteran (avg-%INUC = 37.19 ± 8.22) codon families exhibited much lower levels of conservation.

Neuropterid %INUCs were in some cases very similar (e.g., *trnA, trnH, trnM*) or even surpassed (*trnE, trnG, trnS1*) their lepidopteran counterparts (Figure 9). This finding is striking because it indicates that the level of variation among the three neuropterid orders is very low, taking into account that Lepidoptera have very conserved tRNAs among Insecta [11]. Conversely, Hymenoptera and Coleoptera had diverging codon families. Indeed, all hymenopteran tRNAs showed %INUC values ≤ 47.14, while no coleopteran tRNA exhibited %INUC > 58.21. The %INUC scores indicate a clear-cut discrepancy between the morphological diversity observed in neuropterid orders and the low rate of substitution that characterizes the evolution of their codon families. If the level of divergence is considered a measure of taxonomic diversity, then the %INUC values would suggest that Neuroptera, Megaloptera and Raphidioptera should be treated as members of a single order.

### Comparison of neuropterid *rrnS *and *rrnL *structures

**Figure 10:**
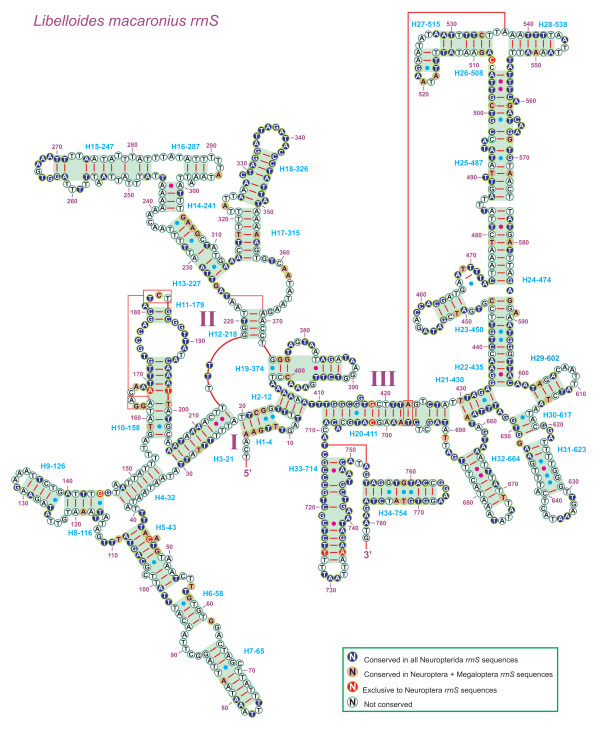
**Secondary structure of *L. macaronius rrnS***. Each helix is numbered progressively from the 5' to the 3' end together with the first nucleotide belonging to the helix itself. Domains are labeled with Roman numerals. Tertiary structures are denoted by boxed bases joined by solid lines. Watson-Crick pairs are joined by dashes. GT pairs are joined by a blue dot, while other non-canonical pairs are connected by a red dot.

The inferred secondary structure model for *rrnS *of *L. macaronius *is provided in Figure 10. This is the first prediction for a neuropterid insect. The overall structure, including three domains and 34 helices (progressively numbered in Figure 10), is largely in agreement with those proposed for other endopterygote orders (i.e. Coleoptera, Diptera, Hymenoptera, and Lepidoptera) [11,17,74,79]. A limited number of non-canonical pairings (e.g., G-A on helix H23-434) were present in the *rrnS *of *L. macaronius*. The multiple alignment of neuropterid *rrnS*s spanned 812 positions and contained 353 conserved (43.47%) and 459 variable (56.53%) positions, respectively. Nucleotide variability among domains and helices was unevenly distributed (Figure 10).

**Figure 11:**
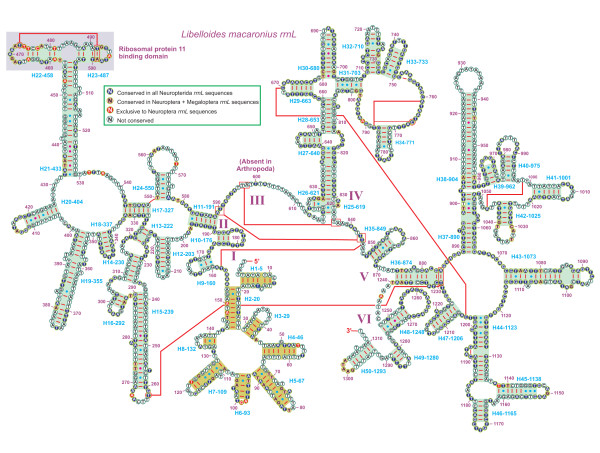
**Secondary structure of *L. macaronius rrnL***. Each helix is numbered progressively from the 5' to the 3' end, followed by the first nucleotide belonging to the helix itself. Domains are labeled with Roman numerals. Tertiary structures are denoted by boxed bases joined by solid lines. Watson-Crick pairs are joined by dashes. GT pairs are joined by a blue dot, while other non-canonical pairs are connected by a red dot.

The inferred secondary structure model for the *rrnL *of *L. macaronius *is provided in Figure 11. This is the first prediction for a neuropterid insect. The structure of this *rrnL *largely overlaps with previously published structures for endopterygote insects (i.e. Coleoptera, Diptera, Hymenoptera, and Lepidoptera) [11,17,74,79]. It presents the five canonical domains (I-II, IV-VI) of insect *rrnLs *that do not contain domain III [74]. In the present paper domain I was fully modeled. This is the first time that a secondary structure has been provided for this domain, which in previous studies has been left unmodeled (e.g., [8,11,56,74,79]). The predicted secondary structure of domain I included eight helices and is consistent among all neuropterid taxa (Additional file 6, Figure S3). The proposed structure can also be fitted to *rrnL *of *D. melanogaster *(Additional file 6, Figure S3), further extending the taxonomic range. Domain I contained a limited number of non-canonical pairings (Additional file 6, Figure S3). More studies will be necessary to corroborate the validity of the new structure presented here. Taking into account the secondary structure of domain I, *L. macaronius rrnL *includes 50 helices. The multiple alignment of neuropterid *rrnL*s extended over 1376 positions and contains 591 conserved (42.95%) and 785 variable sites (57.05%). Conserved nucleotides were unevenly distributed throughout the *rrnL *secondary structure, with highest level of invariable positions located on domain IV and lowest level observed in domains I-II (Figure 11).

### Comparison of neuropterid mtDNA genomic spacers

**Figure 12:**
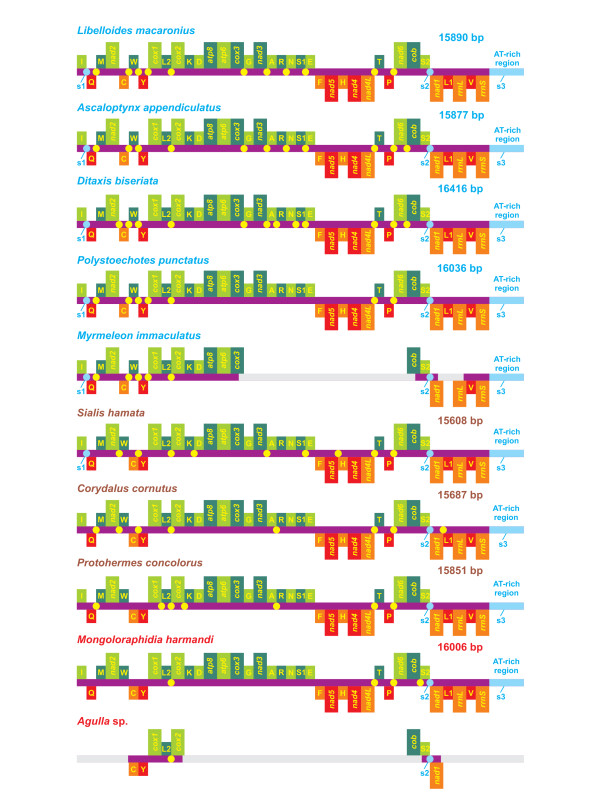
**Distribution of non-coding portions in neuropterid mtDNAs**. The non-coding portions are presented as cyan/yellow dots. s1-s3, spacers 1-3. Gene nomenclature is as in Figures 1 and 2. Genomes, genes and non-coding portions are not drawn to scale, for reasons of clarity. The unknown portions of partial mtDNAs are gray.

The *L. macaronius *mtDNA contained 14 non-coding portions (ncps), extending from 1 to 1049 nucleotides, interspersed through PCGs, tRNAs and *rrnL *and *rrnS *genes (Figure 2). Three of them spanned more than 15 bp and are labeled as spacers s1-s3 in Figures 2 and 12. More or less similar patterns, but not fully overlapping, were observed in other neuropteran mtDNAs, where the ncps varied from a maximum of 15 (*D. biseriata*) to a minimum of 10 (*P. punctatus*), with 13 non-coding portions found in *A. appendiculatus*. The dobsonflies *C. cornutus *and *P. concolorus *exhibited the same number (10) of ncps even if their distributional patterns did not coincide. Conversely, the other megalopteran, *S. hamata*, had 12 ncps. Finally, the raphidiopteran *M. harmandi *exhibited the most compact mtDNA, having only six npcs (Figure 12).

The spacers of *L. macaronius *are described more in detail below. The s1 spacer was located between *trnI *and *trnQ *and spanned 55 bp. Spacer s1 was present in all partly/fully sequenced mtDNAs available for Neuroptera, i.e., *A. appendiculatus, P. punctatus, M. immaculatus *and *D. biseriata *[13,14], present paper)]. It was also found in the megalopteran *S. hamata*, while it was absent in the other megalopterans *C. cornutus *and *P. concolorus *and in the raphidiopteran *M. harmandi *(Figure 12) [13-15]. The *L. macaronius *s1 can be aligned confidently only with the counterpart found in *A. appendiculatus*.

The s1 sequences of *L. macaronius *and *A. appendiculatus *shared a CCCCCC repeat located near the boundary with *trnI *and the CAA(A/G)TTAA(A/C)TAAAT(TA/GT)A(C/T)GCA motif adjacent to *trnQ. A. appendiculatus *and *L. macaronius *belong to the same family, Ascalaphidae. Thus, it seems that s1 sequences, when present, diverge very fast among different families of the same order.

The *L. macaronius *s2 was 20 bp long and was located between *trnS2 *and *nad1*. The s2 spacer was present in all partly or fully sequenced neuropterid mtDNAs (Figure 12) [[13-15], present paper]. The s2 spacer is supposed to contain [13] the binding site for the DmTFF bidirectional transcription termination factor [80]. Spacer s2 is a common feature of insect mtDNAs (e.g., [12]).

The *L. macaronius *s3 spacer was composed of a 1049-bp AT-rich region. The A+T content was 84.46%. This finding implies that s3 contained 10% more A+T than the whole α strand. Conversely, s3 was much less AT-skewed than the complete α strand (0.029 vs. 0.072). Strings of repeated single nucleotides (T)_3-8_, (A)_3-11_, (C)_2-5_, (G)_2-5 _characterized the s3 sequence. Furthermore, the abundance of A/T was mirrored by the presence of repeated motifs containing mostly/only A/T, a common feature of insect control regions [81]. These motifs were represented by shorter identical strings as well as longer consensus patterns sharing 75% of identical positions. The motif AT was the most abundant, occurring 192 times. ATA occurred 70 times, ATAT 29, AATAATTA 5, and ATATAAATA 6. Finally, five distinct A/T-rich 12-mers were repeated twice each (data not shown). When a 75% minimum identity was allowed among repeated motifs, two strings were identified, each containing 37 nucleotides and extending, respectively, from base 551 to base 591 and from 811 to 847. Their consensus sequence was ATATTA (C/T) ATAT (GT/AA) ATA (AT/TG) TA (T/A) TTATTA (A/T) TATATAAAT. Thus, the L. *macaronius *AT-rich region contains several repeated motifs of different sizes. A similar trend also occurs in other neuropterids (data not shown).

Pair-wise as well as multiple alignments between/among neuropterid AT-rich regions were performed by applying very relaxed alignment parameters (i.e., gap-opening = 1 vs. standard 15; and gap-extending = 3 vs. standard 6.6). This strategy was applied to maximize the matches of these fast-evolving genomic portions (see below). This approach allowed us to align quite diverse sequences but did not guarantee that the positional homology principle was retained when the more diverging sequences were compared. The obtained results are summarized in Table 6.

**Table 6:** Pairwise and multilple alignments of neuropterid control regions

ORD		idNP	% ID	ALNL
NEU	*L. macaronius *vs. *A. appendiculatus*	809	71.59%	1130
NEU	*L. macaronius *vs. *P. punctatus*	765	63.80%	1199
NEU	*L. macaronius *vs. *D. biseriata*	845	55.78%	1515
NEU	*L. macaronius *vs. *M. immaculatus*	721	65.13%	1107
NEU	*A. appendiculatus *vs.*M. immaculatus*	731	65.56%	1115
NEU	*A. appendiculatus *vs.*D. biseriata*	863	56.85%	1518
NEU	*A. appendiculatus *vs.*P. punctatus*	787	i64.88%	1213
NEU	*P. punctatus *vs. *D. biseriata*	948	62.00%	1529
NEU	*P. punctatus *vs.*M. immaculatus*	748	63.02%	1187
NEU	*D. biseriata *vs.*M. immaculatus*	813	i53.88%	1509
NEU	Neuroptera multiple alignment	487	30.15%	1615
MEG	*C. cornutus *vs. *P. concolorus*	762	63.50%	1200
MEG	*C. cornutus *vs. *S. hamata*	632	62.27%	1015
MEG	*P. concolorus *vs. *S. hamata*	658	57.52%	1144
MEG	Megaloptera multiple alignment	561	45.91%	1222
	Neuropterida orders multiple alignment	273	14.80%	1845

ORD, order; idNP, identical nucleotide in the alignment position; % ID; percentage of identical nucleotides; ALNL, length of the alignment; NEU Neuroptera, MEG, Megaloptera.

What is immediately evident is that the level of conserved positions dropped rapidly when comparisons were extended above the taxonomic rank of family. Indeed, *L. macaronius *and *A. appendiculatus*, both members of the family Ascalaphidae, shared 71.59% nucleotide identity and a common GC-rich motif TCCCCGGCCCCCCAGGAT located 96 bp downstream of the 5'-end of *rrnS *in *L. macaronius *mtDNA. This motif could be a molecular signature for the whole family, but a better taxon sampling is necessary to assess this point.

When all Neuroptera were considered, the identical positions diminished to 30.15% in the multiple alignment. Thus, a substantial drop in nucleotide identity shared by AT-rich regions occurs at the order level, even permitting very permissive gap costs.

Similarly, *C. cornutus *and *P. concolorus*, both members of the family Corydalidae, shared 63.50% nucleotide identity in their control regions. Conversely, the identity level decreased to 45.91% when all megalopteran AT-rich regions were compared. Finally, the alignment at the interordinal taxonomic rank of neuropterid control regions produced only 14.80% identical positions. Provided that gaps are quite numerous, this result leads to the conclusion that the AT-rich region is a fast-evolving genomic region. This behavior of neuropterid control regions is a common feature of insect AT-rich regions (e.g., [12]) and is shared with other animal groups. In this respect, a well-documented group is represented by mammals (e.g., [82,83]).

The utility of the AT-rich region as a phylogenetic marker should be most effective at low taxonomic rank (family level and below).

## Conclusions

The mtDNA of *L. macaronius*, the fourth genome sequenced for the order Neuroptera, exhibits the peculiar translocation of the *trnC *gene with respect to the ancestral gene order of insects. This structural modification represents an exclusive feature of partly or fully sequenced neuropteran mtDNAs and could be a peculiar genomic marker characterizing the entire order Neuroptera.

The analysis of the AT%, AT-skew and GC-skew parameters, which were performed on a set of 84 holometabolous insect mtDNAs, shows that these characteristics exhibit complex patterns of variations. Such patterns can be linked or completely unlinked in their variation process.

The neuropterid mtDNAs sequenced to date use the standard invertebrate mitochondrial genome. The distribution of codon families in the PCGs located on the α and β strands is influenced by both structural/functional requirements of the corresponding proteins and by the base composition and AT-/GC-skews. The comparison among orthologous neuropterid PCGs shows that different genes have been subject to different rates of molecular evolution and form a pool of markers suitable for different phylogenetic purposes.

The evolution of neuropterid tRNAs seems to have been variable both in terms of sequence conservation and nucleotide substitution processes.

Neuropterid *rrnL *and *rrnS *structures, as demonstrated by the models produced for *L. macaronius*, appear similar to those determined for other insects. In their structural domains, they show diverse levels of conservation, influenced by different rates of substitution.

An important advance in our comprehension of the structure of *rrnL *is the production of a model for its domain I. To our knowledge, this is the first time that such a structural modeling has been attempted for an arthropod *rrnL*.

Neuropterid mtDNAs are punctuated by non-coding portions highly variable in size. Among them, the most extended segment is the control region (AT-rich region), which appears to be a fast-evolving genomic region characterized by short to medium-size repeated motifs/AT-rich patterns.

## Methods

### Sample origin and DNA extraction

The *L. macaronius *specimen used to determine the mtDNA was collected in June 2007 by EN on arid prairies (45°47'35.75"N 13°35'50.63"E, 98 m elevation) located in the Triestine karst along the road connecting San Giovanni al Timavo to Medeazza (Friuli Venezia Giulia region, northeastern Italy).

Total DNA was purified using the Invisorb DNA extraction kit (Invitec). The quality of DNA was assessed through electrophoresis in a 1% agarose gel and staining with SYBR-safe DNA gel stain (Invitrogen).

### PCR amplification and sequencing of *L. macaronius *mtDNA

PCR amplification was performed using a mix of insect universal primers and primers specifically designed against the *L. macaronius *sequences [84,85]. The complete list of successful primers is available upon request. The PCR products were visualized by electrophoresis in a 1% agarose gel and staining with SYBR-safe DNA gel stain. Each PCR product represented by a single electrophoretic band was purified with the ExoSAP-IT kit (Amersham Biosciences) and directly sequenced. Sequencing of both strands was performed at the BMR Genomics service (http://www.bmr-genomics.it/) on automated DNA sequencers mostly employing the primers used for PCR amplification.

### Sequence assembly and annotation

The mtDNA final consensus sequence was assembled using the SeqMan II program from the Lasergene software package (DNAStar, Madison, WI). Gene and strand nomenclature used in this paper were described by Negrisolo et al. [86].

Sequence analysis was performed as follows. Initially, the mtDNA sequence was translated *in silico *into putative proteins using the Transeq program available at the EBI web site. The true identity of these polypeptides was established using the BLAST program available at the NCBI web site [87,88]. Gene boundaries were determined as follows. The 5' ends of PCGs were inferred to be at the first legitimate in-frame start codon (ATN, GTG, TTG, GTT) in the open reading frame that was not located within the upstream gene encoded on the same strand [64,89]. The only exceptions were *atp6 *and *nad4*, which overlap with their upstream genes (*atp8 *and *nad4L*, respectively) in many mtDNAs (e.g., [13]). The PCG terminus was inferred to be at the first in-frame stop codon encountered. When the stop codon was located within the sequence of a downstream gene encoded on the same strand, a truncated stop codon (T or TA) adjacent to the beginning of the downstream gene was designated the termination codon. This codon was thought to be completed by polyadenylation to a complete TAA stop codon after transcript processing. Finally, pair-wise comparisons with orthologous proteins were performed with ClustalW program to better define the limits of PCGs [90].

Irrespective of the real initiation codon, formyl-Met was assumed to be the starting amino acid for all proteins, as previously shown for other mitochondrial genomes [91,92].

The transfer RNA genes were identified using the tRNAscan-SE program or recognized manually as sequences having the appropriate anticodon and capability of folding into the typical cloverleaf secondary structure [64,93].

The boundaries of the ribosomal *rrnL *gene were assumed to be delimited by the ends of the *trnV*-*trnL1 *pair. The 3' end of the *rrnS *gene was assumed to be delimited by the start of *trnV*, while the 5' end was determined through comparison with orthologous genes of other previously sequenced neuropterid insect mtDNAs.

The published neuropterid genomes were re-annotated using the criteria listed above. This approach led us to propose different start/end positions for some genes.

### Genomic analysis

Nucleotide composition was calculated with the EditSeq program included in the Lasergene software package. GC-skew = (G-C)/(G+C) and AT-skew = (A-T)/(A+T) were used to measure the base-compositional difference between the different strands or between genes coded on the alternative strands [94]. The prediction of the genetic code used by each mtDNA was performed using the GenDecoder web server [67,68].

Relative synonymous codon usage (RSCU) values were calculated with MEGA 4 program [95].

Codon usage in neuropterid mtDNAs was studied by calculating the effective number of codon used index (ENC) with the INCA 2.1 program [71,96]. Multiple alignments of genes were produced with the ClustalW algorithm implemented in MEGA 4 [90,95]. Both p-distances and the numbers of different nucleotides in pair-wise comparisons were calculated with the PAUP* program [97].

Sequence motifs in the AT-rich region were identified using the Spectral Repeat Finder program [98]. Not only simple motifs were searched but also longer consensus patterns with a minimum match of 75% among different strings.

### Testing for phylogenetic signal and saturation of the substitution process

An a priori estimation of the phylogenetic signal present in the multiple alignments was performed by maximum likelihood mapping [70]. The phylogenetic signal was evaluated using the TREEPUZZLE 5.2 program [99].

For the nucleotide sequences, the level of saturation of the substitution process was estimated by plotting uncorrected p-distances (based on observed substitutions) against GTR+I+G estimated distances for multiple alignment of single PCGs as well as concatenated PCGs. In the case of amino acid sequences, the level of saturation was estimated by plotting uncorrected p-distances against mtREV24/JTT + G models implemented in MEGA4 and TREEPUZZLE 5.2. After fitting a regression line, its slope (m) was used as a measure of saturation. If m = 1, no saturation is inferred, while for m <<1, a high level of saturation has occurred.

### *rrnL *and *rrnS *homology modeling

Secondary structures of *rrnL *and *rrnS *were produced through homology modeling using as templates published structures of *Apis mellifera, Drosophila melanogaster *and *Drosophila virilis *[74,79].

## Abbreviations

mtDNA: mitochondrial DNA; *atp6 *and *atp8*: ATP synthase subunits 6 and 8; *cob*: apocytochrome b; *cox1-3*: cytochrome c oxidase subunits 1-3; *nad1*-*6 *and *nad4L*: NADH dehydrogenase subunits 1-6 and 4L; *rrnS *and *rrnL*: small and large subunit ribosomal RNA (rRNA) genes; *trnX*: transfer RNA (tRNA) genes: where X is the one-letter abbreviation of the corresponding amino acid; s1-s3: mitochondrial genomic spacers; A+T region: the putative control region; PCG: protein-coding gene; RSCU: relative synonymous codon usage; ENC: effective number of codons used; nt: nucleotides; bp: base pairs.

## Authors' contributions

EN and TP designed and coordinated all experiments. EN and MB conducted the molecular experiments. EN performed the genomic analyses and wrote the first draft of the manuscript. All authors contributed the final version of the manuscript and approved it.

## Supplementary Material

Additional file 1 Table S1: Subdivision of endopterygotan mtDNAs in clusters based on A+T content (T1a), AT-skew T1b), and GC-skew/T1c9 respectively. Click here for file

Additional file 2 Table S2: A+T%, AT-skew, G+C%, GC-skew, for whole genome α strand; pooled α + β strands PCGs; pooled-α strand PCGs and pooled-β strand PCGs. Click here for file

Additional file 3 **Figure S1: Relative Synonymous Codon Usage (RSCU) in neuropterid pooled α-strand protein-coding genes**. Codon families are provided on the x axis. Red-colored codon, codon not present in the pooled genes. Click here for file

Additional file 4 **Figure S2: Relative Synonymous Codon Usage (RSCU) in neuropterid pooled β-strand protein-coding genes**. Codon families are provided on the x axis. Red-colored codon, codon not present in the pooled genes. Click here for file

Additional file 5 Table S3: Summary of multiple alignments of tRNA families in neuropterid mtDNAs. Click here for file

Additional file 6 **Figure S3: Domain I of *rrnL *in neuropterid species and in *Drosophila melanogaster***. Green background, conserved nucleotide in the pair-wise alignment with *L. macaronius*. Click here for file

## References

[B1] Boore JL Animal mitochondrial genomes Nucleic Acids Res 1999 27 1767 1780 10.1093/nar/27.8.1767 10101183 PMC148383

[B2] Shao R Kirkness EF Barker SC The single mitochondrial chromosome typical of animals has evolved into 18 minichromosomes in the human body louse, *Pediculus humanus* Genome Res 2009 19 904 912 10.1101/gr.083188.108 19336451 PMC2675979

[B3] Boyce TM Zwick ME Aquadro CF Mitochondrial DNA in the bark weevils: size, structure and heteroplasmy Genetics 1989 123 825 836 2612897 10.1093/genetics/123.4.825 PMC1203892

[B4] Boore, JL Collins TM Stanton D Daehler LL Brown WM Deducing the pattern of arthropod phylogeny from mitochondrial DNA rearrangements Nature 1995 376 163 165 10.1038/376163a0 7603565

[B5] Boore JL Lavrov DV Brown WM Gene translocation links insects and crustaceans Nature 1998 392 667 668 10.1038/33577 9565028

[B6] Clary DO Wolstenholme DR The mitochondrial DNA molecular of *Drosophila yakuba*: nucleotide sequence, gene organization, and genetic code J Mol Evol 1985 22 252 271 10.1007/BF02099755 3001325

[B7] Cameron SL Beckenbach AT Dowton M Whiting MF Evidence from mitochondrial genomics on interordinal relationships in insects Arthropods Syst Phyl 2006 64 27 34

[B8] McMahon DP Hayward A Kathirithamby J The mitochondrial genome of the 'twisted-wing parasite' *Mengenilla australiensis *(Insecta, Strepsiptera): a comparative study BMC Genomics 2009 10 603 1 15 20003419 10.1186/1471-2164-10-603 PMC2800125

[B9] Beard CB Hamm DM Collins FH The mitochondrial genome of the mosquito *Anopheles gambiae*: DNA sequence, genome organization, and comparisons with mitochondrial sequences of other insects Insect Mol Biol 1993 2 103 124 10.1111/j.1365-2583.1993.tb00131.x 9087549

[B10] Mitchell SE Cockburn AF Seawright JA The mitochondrial genome of *Anopheles quadrimaculatus *species A: complete nucleotide sequence and gene organization Genome 1993 36 1058 1073 10.1139/g93-141 8112570

[B11] Cameron SL Whiting MF The complete mitochondrial genome of the tobacco hornworm, *Manduca sexta*, (Insecta: Lepidoptera: Sphingidae), and an examination of mitochondrial gene variability within butterflies and moths Gene 2008 408 112 123 10.1016/j.gene.2007.10.023 18065166

[B12] Salvato P Simonato M Battisti A Negrisolo E The complete mitochondrial genome of the bag-shelter moth *Ochrogaster lunifer *(Lepidoptera, Notodontidae) BMC Genomics 2008 9 331 1 15 18627592 10.1186/1471-2164-9-331 PMC2488359

[B13] Beckenbach AT Stewart JB Insect mitochondrial genomics 3: the complete mitochondrial genome sequences of representatives from two neuropteroid orders: a dobsonfly (order Megaloptera) and a giant lacewing and an owlfly (order Neuroptera) Genome 2009 52 31 38 10.1139/G08-098 19132069

[B14] Cameron SL Sullivan A Song H Miller KB A mitochondrial genome phylogeny of the Neuropterida (lace-wings, alderflies and snakeflies) and their relationship to the other holometabolous insect orders Zool Scr 2009 38 575 590 10.1111/j.1463-6409.2009.00392.x

[B15] Hua J Li M Dong P Xie Q Bu W The mitochondrial genome of *Protohermes concolorus *Yang et Yang 1988 (Insecta: Megaloptera: Corydalidae) Mol Biol Rep 2009 36 1757 1765 10.1007/s11033-008-9379-0 18949579

[B16] Sheffield NC Song H Cameron SL Whiting MF Nonstationary evolution and compositional heterogeneity in beetle mitochondrial phylogenomics Syst Biol 2009 58 381 394 10.1093/sysbio/syp037 20525592

[B17] Sheffield NC Song H Cameron SL Whiting MF A comparative analysis of mitochondrial genomes in Coleoptera (Arthropoda: Insecta) and genome descriptions of six new beetles Mol Biol Evol 2008 25 2499 2509 10.1093/molbev/msn198 18779259 PMC2568038

[B18] Hong MY Jeong HC Kim MJ Jeong HU Lee SH Kim I Complete mitogenome sequence of the jewel beetle, *Chrysochroa fulgidissima *(Coleoptera: Buprestidae) Mitochondrial DNA 2009 20 46 60 19444700 10.1080/19401730802644978

[B19] Stewart JB Beckenbach AT Phylogenetic and genomic analysis of the complete mitochondrial DNA sequence of the spotted asparagus beetle *Crioceris duodecimpunctata* Mol Phylogent Evol 2003 26 513 526 10.1016/S1055-7903(02)00421-9 12644408

[B20] Kim KG Hong MY Kim MJ Im HH Kim MI Bae CH Seo SJ Lee SH Kim I Complete mitochondrial genome sequence of the yellow-spotted long-horned beetle *Psacothea hilaris *(Coleoptera: Cerambycidae) and phylogenetic analysis among coleopteran insects Mol Cells 2009 27 429 441 10.1007/s10059-009-0064-5 19390824

[B21] Bae JS Kim I Sohn HD Jin BR The mitochondrial genome of the firefly, *Pyrocoelia rufa*: complete DNA sequence, genome organization, and phylogenetic analysis with other insects Mol Phylogent Evol 2004 32 978 985 10.1016/j.ympev.2004.03.009 15288070

[B22] Arnoldi FG Ogoh K Ohmiya Y Viviani VR Mitochondrial genome sequence of the Brazilian luminescent click beetle *Pyrophorus divergens *(Coleoptera: Elateridae): mitochondrial genes utility to investigate the evolutionary history of Coleoptera and its bioluminescence Gene 2007 405 1 9 10.1016/j.gene.2007.07.035 17942246

[B23] Li X Ogoh K Ohba N Liang X Ohmiya Y Mitochondrial genomes of two luminous beetles, *Rhagophthalmus lufengensis *and *R. ohbai *(Arthropoda, Insecta, Coleoptera) Gene 2007 392 196 205 10.1016/j.gene.2006.12.017 17300880

[B24] Friedrich M Muqim N Sequence and phylogenetic analysis of the complete mitochondrial genome of the flour beetle *Tribolium castanaeum* Mol Phylogent Evol 2003 26 502 512 10.1016/S1055-7903(02)00335-4 12644407

[B25] Yu DJ Xu L Nardi F Li JG Zhang RJ The complete nucleotide sequence of the mitochondrial genome of the oriental fruit fly, *Bactrocera dorsalis *(Diptera: Tephritidae) Gene 2007 396 66 74 10.1016/j.gene.2007.02.023 17433576

[B26] Nardi F Carapelli A Dallai R Frati F The mitochondrial genome of the olive fly *Bactrocera oleae*: two haplotypes from distant geographical locations Insect Mol Biol 2003 12 605 611 10.1046/j.1365-2583.2003.00445.x 14986921

[B27] Spanos L Koutroumbas G Kotsyfakis M Louis C The mitochondrial genome of the mediterranean fruit fly, *Ceratitis capitata* Insect Mol Biol 2000 9 139 144 10.1046/j.1365-2583.2000.00165.x 10762421

[B28] Junqueira AC Lessinger AC Torres TT Da Silva FR Vettore AL Arruda P Azeredo Espin AM The mitochondrial genome of the blowfly *Chrysomya chloropyga *(Diptera: Calliphoridae) Gene 2004 339 7 15 15363841 10.1016/j.gene.2004.06.031

[B29] Lessinger AC Martins Junqueira AC Lemos TA Kemper EL da Silva FR Vettore AL Arruda P Azeredo-Espin AM The mitochondrial genome of the primary screwworm fly *Cochliomyia hominivorax *(Diptera: Calliphoridae) Insect Mol Biol 2000 9 521 529 10.1046/j.1365-2583.2000.00215.x 11029671

[B30] Cameron SL Lambkin CL Barker SC Whiting MF A mitochondrial genome phylogeny of Diptera: whole genome sequence data accurately resolve relationships over broad timescales with high precision Syst Entomol 2007 32 40 59 10.1111/j.1365-3113.2006.00355.x

[B31] Ballard JW Comparative genomics of mitochondrial DNA in members of the *Drosophila melanogaster *subgroup J Mol Evol 2000 51 48 63 10903372 10.1007/s002390010066

[B32] Lewis DL Farr CL Kaguni LS *Drosophila melanogaster *mitochondrial DNA: completion of the nucleotide sequence and evolutionary comparisons Insect Mol Biol 1995 4 263 278 10.1111/j.1365-2583.1995.tb00032.x 8825764

[B33] Oliveira MT Grande Barau J Martins Junqueira AC Feijão PC Coelho da Rosa A Feix Abreu C Azeredo-Espin AML Lessinger AC Structure and evolution of the mitochondrial genomes of *Haematobia irritans *and *Stomoxys calcitrans*: The Muscidae (Diptera: Calyptratae) perspective Mol Phylogenet Evol 2008 48 850 857 10.1016/j.ympev.2008.05.022 18621550

[B34] Stevens JR West H Wall R Mitochondrial genomes of the sheep blowfly, *Lucilia sericata*, and the secondary blowfly, *Chrysomya megacephala* Med Vet Entomol 2008 22 89 91 10.1111/j.1365-2915.2008.00710.x 18380659

[B35] Beckenbach AT Joy JB Evolution of the Mitochondrial Genomes of Gall Midges (Diptera:Cecidomyiidae): Rearrangement and Severe Truncation of tRNA Genes Genome Biol Evol 2009 1 278 287 20333197 10.1093/gbe/evp027 PMC2817422

[B36] Cameron SL Dowton M Castro LR Ruberu K Whiting MF Austin AD Diement K Stevens J Mitochondrial genome organization and phylogeny of two vespid wasps Genome 2008 51 800 808 10.1139/G08-066 18923531

[B37] Crozier RH Crozier YC The mitochondrial genome of the honeybee *Apis mellifera*: complete sequence and genome organization Genetics 1993 133 97 117 8417993 10.1093/genetics/133.1.97 PMC1205303

[B38] Cha SY Yoon HJ Lee EM Yoon MH Hwang JS Jin BR Han YS Kim I The complete nucleotide sequence and gene organization of the mitochondrial genome of the bumblebee, *Bombus ignitus *(Hymenoptera: Apidae) Gene 2007 392 206 220 10.1016/j.gene.2006.12.031 17321076

[B39] Dowton M Cameron SL Dowavic JI Austin AD Whiting MF Characterization of 67 mitochondrial tRNA gene rearrangements in the Hymenoptera suggests that mitochondrial tRNA gene position is selectively neutral Mol Biol Evol 2009 26 1607 1617 10.1093/molbev/msp072 19359443

[B40] Wei SJ Shi M He JH Sharkey M Chen XX The complete mitochondrial genome of *Diadegma semiclausum *(hymenoptera: ichneumonidae) indicates extensive independent evolutionary events Genome 2009 52 308 319 10.1139/G09-008 19370087

[B41] Wei SJ Tang P Zheng LH Shi M Chen XX The complete mitochondrial genome of *Evania appendigaster *(Hymenoptera: Evaniidae) has low A+T content and a long intergenic spacer between *atp8 *and *atp6* Mol Biol Rep 2010 37 1931 1942 10.1007/s11033-009-9640-1 19655273 PMC2831182

[B42] Silvestre D Dowton M Arias MC The mitochondrial genome of the stingless bee *Melipona bicolor *(Hymenoptera, Apidae, Meliponini): Sequence, gene organization and a unique tRNA translocation event conserved across the tribe Meliponini Genet Mol Biol 2008 31 451 460 10.1590/S1415-47572008000300010

[B43] Hu J Zhang D Hao J Huang D Cameron S Zhu C The complete mitochondrial genome of the yellow coaster, *Acraea issoria *(Lepidoptera: Nymphalidae: Heliconiinae: Acraeini): sequence, gene organization and a unique tRNA translocation event Mol Biol Rep 2010 37 3431 3438 10.1007/s11033-009-9934-3 20091125

[B44] Lee ES Shin KS Kim MS Park H Cho S Kim CB The mitochondrial genome of the smaller tea tortrix *Adoxophyes honmai *(Lepidoptera: Tortricidae) Gene 2006 373 52 57 16488090 10.1016/j.gene.2006.01.003

[B45] Liu Y Li Y Pan M Dai F Zhu X LuC Xiang Z The complete mitochondrial genome of the Chinese oak silkmoth, *Antheraea pernyi *(Lepidoptera: Saturniidae) Acta Biochim Biophys Sin 2008 40 693 703 18685785

[B46] Kim SR Kim MI Hong MY Kim KY Kang PD Hwang JS Han YS Jin BR Kim I The complete mitogenome sequence of the Japanese oak silkmoth, *Antheraea yamamai *(Lepidoptera: Saturniidae) Mol Biol Rep 2009 36 1871 1880 10.1007/s11033-008-9393-2 18979227

[B47] Hong G Jiang S Yu M Yang Y Li F Xue F Wei Z The complete nucleotide sequence of the mitochondrial genome of the cabbage butterfly, *Artogeia melete *(Lepidoptera: Pieridae) Acta Biochim Biophys Sin 2009 41 446 455 10.1093/abbs/gmp030 19499147

[B48] Yukuhiro K Sezutsu H Itoh M Shimizu K Banno Y Significant levels of sequence divergence and gene rearrangements have occurred between the mitochondrial genomes of the wild mulberry silkmoth, *Bombyx mandarina *and its close relative, the domesticated silkmoth, *Bombyx mori* Mol Biol Evol 2002 19 1385 1389 12140251 10.1093/oxfordjournals.molbev.a004200

[B49] Kim I Lee EM Seol KY Yun EY Lee YB Hwang JS Jin BR The mitochondrial genome of the Korean hairstreak, *Coreana raphaelis *(Lepidoptera: Lycaenidae) Insect Mol Biol 2006 15 217 225 10.1111/j.1365-2583.2006.00630.x 16640732

[B50] Jiang ST Hong GY Yu M Li N Yang Y Liu YQ Wei ZJ Characterization of the complete mitochondrial genome of the giant silkworm moth, *Eriogyna pyretorum *(Lepidoptera: Saturniidae) Int J Biol Sci 2009 5 351 365 19471586 10.7150/ijbs.5.351 PMC2686093

[B51] Yang L Wei ZJ Hong GY Jiang ST Wen LP The complete nucleotide sequence of the mitochondrial genome of *Phthonandria atrilineata *(Lepidoptera: Geometridae) Mol Biol Rep 2009 36 1441 1449 10.1007/s11033-008-9334-0 18696255

[B52] Hong MY Lee EM Jo YH Park HC Kim SR Hwang JS Jin BR Kang PD Kim KG Han YS Kim I Complete nucleotide sequence and organization of the mitogenome of the silk moth *Caligula boisduvalii *(Lepidoptera: Saturniidae) and comparison with other lepidopteran insects Gene 2008 413 49 57 10.1016/j.gene.2008.01.019 18337026

[B53] Wiegmann BM Trautwein MD Kim JW Cassel BK Bertone MA Winterton SL Yeates DK Single-copy nuclear genes resolve the phylogeny of the holometabolous insects BMC Biology 2009 7 34 1 16 19552814 10.1186/1741-7007-7-34 PMC2709105

[B54] Wei SJ Shi M Sharkey MJ van Achterberg C Chen XX Comparative mitogenomics of Braconidae (Insecta: Hymenoptera) and the phylogenetic utility of mitochondrial genomes with special reference to Holometabolous insects BMC Genomics 2010 11 371 1 16 20537196 10.1186/1471-2164-11-371 PMC2890569

[B55] Aspöck U Plant JD Nemeschkal HL Cladistic analysis of Neuroptera and their systematic position within Neuropterida (Insecta: Holometabola: Neuropterida: Neuroptera) Syst Entomol 2001 26 73 86 10.1046/j.1365-3113.2001.00136.x

[B56] Haring E Aspöck U Phylogeny of the Neuropterida: a first molecular approach Syst Entomol 2004 29 415 430 10.1111/j.0307-6970.2004.00263.x

[B57] Winterton SL Hardy NB Wiegmann BM On wings of lace: phylogeny and Bayesian divergence time estimates of Neuropterida (Insecta) based on morphological and molecular data Syst Entomol 2010 35 349 378 10.1111/j.1365-3113.2010.00521.x

[B58] Carapelli A Vannini L Nardi F Boore JL Beani L Dallai R Frati F The mitochondrial genome of the entomophagous endorparasite *Xenos vesparum *(Insecta: Strepsiptera) Gene 2006 376 248 259 10.1016/j.gene.2006.04.005 16766140

[B59] Castro LR Dowton M The position of the Hymenoptera within the Holometabola as inferred from the mitochondrial genome of *Perga condei *(Hymenoptera: Symphyta: Pergidae) Mol Phylogenet Evol 2005 34 469 479 10.1016/j.ympev.2004.11.005 15683922

[B60] Castro LR Ruberu K Dowton M Mitochondrial genomes of *Vanhornia eucnemidarum *(Apocrita: Vanhorniidae) and *Primeuchroeus *spp. (Aculeata: Chrysididae): Evidence of rearranged mitochondrial genomes within the Apocrita (Insecta: Hymenoptera) Genome 2006 49 752 766 10.1139/G06-030 16936784

[B61] Dowton M Relationships among the cyclostome braconid (Hymenoptera: Braconidae) subfamilies inferred from a mitochondrial tRNA gene rearrangement Mol Phylogenet Evol 1999 11 283 287 10.1006/mpev.1998.0580 10191072

[B62] Dowton M Austin AD Evolutionary dynamics of a mitochondrial rearrangement ''hotspot'' in the Hymenoptera Mol Biol Evol 1999 16 298 309 10028295 10.1093/oxfordjournals.molbev.a026111

[B63] Dowton M Castro LR Campbell SL Bargon SD Austin AD Frequent mitochondrial gene rearrangements at the hymenopteran *nad3-nad5 *junction J Mol Evol 2003 56 517 526 10.1007/s00239-002-2420-3 12698290

[B64] Wolstenholme DR Animal mitochondrial DNA: structure and evolution Int Rev Cytol 1992 141 173 216 1452431 10.1016/s0074-7696(08)62066-5

[B65] Montooth KL Abt DN Hofmann JW Rand DM Comparative genomics of Drosophila mtDNA: novel features of conservation and change across functional domains and lineages J Mol Evol 2009 69 94 114 10.1007/s00239-009-9255-0 19533212 PMC2895726

[B66] Stewart JB Beckenbach AT Characterization of mature mitochondrial transcripts in Drosophila, and the mplications for the tRNA punctuation model in arthropods Gene 2009 445 49 57 10.1016/j.gene.2009.06.006 19540318

[B67] Abascal F Posada D Knight RD Zardoya R Parallel evolution of the genetic code in arthropod mitochondrial genomes PLoS Biol 2006 4 e127 (1-8) 10.1371/journal.pbio.0040127 16620150 PMC1440934

[B68] Abascal F Zardoya R Posada D GenDecoder: genetic code prediction for metazoan mitochondria Nucleic Acids Res 2006 34 W389 W393 10.1093/nar/gkl044 16845034 PMC1538875

[B69] Mishmar D Ruiz-Pesini E Mondragon-Palomino M Procaccio V Gaut B Wallace DC Adaptive selection of mitochondrial complex I subunits during primate radiation Gene 2006 378 11 18 16828987 10.1016/j.gene.2006.03.015

[B70] Strimmer K von Haeseler A Likelihood-mapping: a simple method to visualize phylogenetic content of a sequence alignment Proc Natl Acad Sci USA 1997 94 6815 6819 10.1073/pnas.94.13.6815 9192648 PMC21241

[B71] Supek F Vlahovičekl K INCA: synonymous codon usage analysis and clustering by means of self-organizing map Bioinformatics 2004 20 2329 2330 10.1093/bioinformatics/bth238 15059815

[B72] Negrisolo E Minelli A Valle G The mitochondrial genome of the house centipede *Scutigera *and myriapod monophyly vs. paraphyly Mol Biol Evol 2004 21 770 780 10.1093/molbev/msh078 14963096

[B73] Lavrov DV Brown WM Boore JL A novel type of RNA editing occurs in the mitochondrial tRNAs of the centipede *Lithobius forficatus* Proc Natl Acad Sci USA 2000 97 13738 13742 10.1073/pnas.250402997 11095730 PMC17645

[B74] Cannone JJ Subramanian S Schnare MN Collett JR D'Souza LM Du Y Feng B Lin N Madabusi LV Müller KM Pande N Shang Z Yu N Gutell RR The Comparative RNA Web (CRW) Site: an online database of comparative sequence and structure information for ribosomal, intron, and other RNAs BMC Bioinformatics 2002 3 2 1 31 11869452 10.1186/1471-2105-3-2 PMC65690

[B75] Coleman AW ITS2 is a double-edged tool for eukaryote evolutionary comparisons Trends Genet 2003 19 370 375 10.1016/S0168-9525(03)00118-5 12850441

[B76] Kim I Cha SY Yoon MH Hwang JS Lee SM Sohn HD Jin BR The complete nucleotide sequence and gene organization of the mitochondrial genome of the oriental mole cricket, *Gryllotalpa orientalis *(Orthoptera: Gryllotalpidae) Gene 2005 353 155 168 10.1016/j.gene.2005.04.019 15950403

[B77] Fenn JD Song H Cameron SL Whiting MF A preliminary mitochondrial genome phylogeny of Orthoptera (Insecta) and approaches to maximizing phylogenetic signal found within mitochondrial genome data Mol Phylogenet Evol 2008 49 59 68 10.1016/j.ympev.2008.07.004 18672078

[B78] Miya M Satoh TP Nishida M The phylogenetic position of toadfishes (order Batrachoidiformes) in the higher ray-finned fish as inferred from partitioned Bayesian analysis of 102 whole mitochondrial genome sequences Biol J Linn Soc Lond 2005 85 289 306 10.1111/j.1095-8312.2005.00483.x

[B79] Gillespie JJ Johnston JS Cannone JJ Gutell RR Characteristics of the nuclear (18S, 5.8S, 28S and 5S) and mitochondrial (12S and 16S) rRNA genes of *Apis mellifera *(Insecta: Hymenoptera): structure, organization, and retrotransposable elements Insect Mol Biol 2006 15 657 686 10.1111/j.1365-2583.2006.00689.x 17069639 PMC2048585

[B80] Roberti M Polosa PL Bruni F Musicco C Gadaleta MN Cantatore P DmTTF, a novel mitochondrial transcription termination factor that recognises two sequenze in *Drosophila melanogaster *mitochondrial DNA Nucleic Acid Res 2003 31 1597 1604 10.1093/nar/gkg272 12626700 PMC152874

[B81] Zhang DX Hweitt GM Insect mitochondrial control region: a review of its structure, evolution and usefulness in evolutionary studies Biochem Syst Ecol 1997 25 99 120 10.1016/S0305-1978(96)00042-7

[B82] Larizza A Pesole G Reyes A Sbisà E Saccone C Lineage specificity of the evolutionary dynamics of the mtDNA D-Loop region in rodents J Mol Evol 2002 54 145 155 10.1007/s00239-001-0063-4 11821908

[B83] Larizza A Makalowski W Pesole G Saccone C Evolutionary dynamics of mammalian mRNA untranslated regions by comparative analysis of orthologous human, artiodactyl and rodent gene pairs Comput Chem 2002 26 479 490 12144177 10.1016/s0097-8485(02)00009-8

[B84] Simon C Frati F Beckenbach A Crespi B Liu H Flook P Evolution, weighting, and phylogenetic utility of mitochondrial gene sequences and a compilation of conserved polymerase chain reaction primers Ann Entomol Soc Am 1994 87 651 704

[B85] Simon C Buckley TR Frati F Stewart JB Beckenbach AT Incorporating molecular evolution into phylogenetic analysis, and a new compilation of conserved polymerase chain reaction primers for animal mitochondrial DNA Annu Rev Ecol Evol Syst 2006 37 545 579 10.1146/annurev.ecolsys.37.091305.110018

[B86] Negrisolo E Minelli A Valle G Extensive gene order rearrangement in the mitochondrial genome of the centipede *Scutigera coleoptrata* J Mol Evol 2004 58 413 423 10.1007/s00239-003-2563-x 15114420

[B87] Altschul SF Gish W Miller W Myers EW Lipman DJ Basic local alignment search tool J Mol Biol 1990 215 403 410 2231712 10.1016/S0022-2836(05)80360-2

[B88] Tatusova TA Madden TL BLAST 2 Sequences, a new tool for comparing protein and nucleotide sequences FEMS Microbiol Lett 1999 174 247 250 10.1111/j.1574-6968.1999.tb13575.x 10339815

[B89] Lavrov DV Boore JL Brown WM The complete mitochondrial DNA sequence of the horseshoe crab *Limulus polyphemus* Mol Biol Evol 2000 17 813 824 10779542 10.1093/oxfordjournals.molbev.a026360

[B90] Thompson JD Higgins DG Gibson TJ Clustal-W - improving the sensitivity of progressive multiple sequence alignment through sequence weighting, position-specific gap penalties and weight matrix choice Nucleic Acids Res 1994 22 4673 4680 10.1093/nar/22.22.4673 7984417 PMC308517

[B91] Smith AE Marcker KA N-formylmethionyl transfer RNA in mitochondria from yeast and rat liver J Mol Biol 1968 38 241 243 10.1016/0022-2836(68)90409-9 5760639

[B92] Fearnley IM Walker JE Initiation codons in mammalian mitochondria: differences in genetic code in the organelle Biochemistry 1987 26 8247 8251 10.1021/bi00399a034 2964865

[B93] Lowe TM Eddy SR tRNAscan-SE: a program for improved detection of transfer RNA genes in genomic sequence Nucleic Acids Res 1997 25 955 964 10.1093/nar/25.5.955 9023104 PMC146525

[B94] Perna NT Kocher TD Patterns of nucleotide composition at fourfold degenerate sites of animal mitochondrial genomes J Mol Evol 1995 41 353 358 10.1007/BF01215182 7563121

[B95] Tamura K Dudley J Nei M Kumar S MEGA4: Molecular Evolutionary Genetics Analysis (MEGA) Software Version 4.0 Mol Biol Evol 2007 24 1596 1599 10.1093/molbev/msm092 17488738

[B96] Wright F The 'effective number of codons' used in a gene Gene 1990 87 23 29 10.1016/0378-1119(90)90491-9 2110097

[B97] Swofford DL PAUP*, Phylogenetic Analysis using Parsimony (*and other methods). Version 4.10 2002 Sunderland MA: Sinauer Associates

[B98] Sharma D Issac B Raghava GPS Ramaswamy R Spectral Repeat Finder (SRF): identification of repetitive sequences using Fourier transformation Bioinformatics 2004 20 1405 1412 10.1093/bioinformatics/bth103 14976032

[B99] Schmidt HA Strimmer K Vingron M von Haeseler A TREE-PUZZLE: maximum likelihood phylogenetic analysis using quartets and parallel computing Bioinformatics 2002 18 502 504 10.1093/bioinformatics/18.3.502 11934758

