# Supplementary material for: The mitochondrial genome of the ascalaphid owlfly Libelloides macaronius and comparative evolutionary mitochondriomics of neuropterid insects
Source: BMC Genomics. 2011 May 10;12:221. doi: 10.1186/1471-2164-12-221 (PMC3115881; doi:10.1186/1471-2164-12-221)
Supplement: Additional file 1 — Table S1: Subdivision of endopterygotan mtDNAs in clusters based on A+T content (T1a), AT-skew T1b), and GC-skew/T1c9 respectively. [file 1471-2164-12-221-S1.PDF]

Additional file 1, Negrisolo et al. 2011

Supporting Table S1a. Clusters of A+T%

ORD	N	TAXON	A+T%	CL	n
COL	22	*Tetraphalerus bruchi*	66,99		
COL	11	*Lucanus mazama*	67,11		
COL	4	*Apatides fortis*	67,19	**A1**	**5**
COL	1	*Acmaeodera* sp	68,41		
COL	17	*Pyrophorus divergens*	69,44		
COL	7	*Chrysochroa fulgidissima*	69,92		
COL	24	*Tribolium castaneum*	71,68		
COL	13	*Mordella atrata*	71,94		
DIP	31	*Bactrocera oleae*	72,63		
COL	2	*Adelium* sp	72,71		
DIP	32	*Bactrocera papayae*	73,52		
DIP	29	*Bactrocera carambolae*	73,55	**A2**	**11**
DIP	30	*Bactrocera dorsalis*	73,58		
DIP	33	*Bactrocera philippinensis*	73,63		
DIP	51	*Trichophthalma punctata*	73,96		
NEU	76	*Libelloides macaronius*	74,5		
MEG	80	*Corydalus cornutus*	74,9		
COL	9	*Cyphon* sp.	75,17		
NEU	78	*Ascaloptynx appendiculatus*	75,57		
COL	20	*Rhopaea magnicornis*	75,59		
MEG	81	*Protohermes concolorus*	75,83		
HYM	59	*Orussus occidentalis*	76,21		
DIP	40	*Drosophila litoralis*	76,24		
MCP	84	*Neopanorpa pulchra*	76,38		
COL	14	*Priasilpha obscura*	76,5		
COL	15	*Psacothea hilaris*	76,63		
DIP	35	*Chrysomya putoria*	76,7		
COL	6	*Chauliognathus opacus*	76,86		
COL	8	*Crioceris duodecimpunctata*	76,89		
DIP	36	*Cochliomyia hominivorax*	76,9		
COL	10	*Hydroscapha granulum*	77,29		
DIP	28	*Anopheles quadrimaculatus A*	77,36		
DIP	37	*Culicoides arakawae*	77,36		
COL	16	*Pyrocoelia rufa*	77,41		
DIP	34	*Ceratitis capitata*	77,48		
DIP	27	*Anopheles gambiae*	77,56		
DIP	43	*Drosophila sechellia*	77,57		
DIP	47	*Lucilia sericata*	77,61		
DIP	41	*Drosophila mauritiana*	77,71	**A3**	**43**
HYM	57	*Evania appendigaster*	77,77		
DIP	39	*Dermatobia hominis*	77,82		
LEP	73	*Ochrogaster lunifer*	77,84		
DIP	38	*Cydistomyia duplonotata*	77,93		
DIP	44	*Drosophila simulans*	77,93		
COL	12	*Macrogyrus oblongus*	78		
COL	3	*Anoplophora glabripennis*	78,31		
MEG	82	*Sialis hamata*	78,32		
DIP	45	*Drosophila yakuba*	78,59		
NEU	77	*Polystoechotes punctatus*	78,96	**A3**	
DIP	25	*Aedes aegypti*	79		
COL	5	*Chaetosoma scaritides*	79,04		
DIP	46	*Haematobia irritans*	79,07		
COL	19	*Rhagophthalmus ohbai*	79,15		
COL	23	*Trachypachus holmbergi*	79,45		
DIP	26	*Aedes albopictus*	79,54		
COL	18	*Rhagophthalmus lufengensis*	79,63		
LEP	61	*Acraea issoria*	79,76		
LEP	65	*Artogeia melete*	79,78		
NEU	79	*Ditaxis biseriata*	79,79		
LEP	71	*Lymantria dispar*	79,88		
LEP	69	*Diatraea saccharalis*	80,02		
HYM	60	*Vanhornia eucnemidarum*	80,11		
LEP	63	*Antheraea pernyi*	80,16		
LEP	64	*Antheraea yamamai*	80,29		
RPH	83	*Mongoloraphidia harmandi*	80,31		
LEP	62	*Adoxophyes honmai*	80,39		
HYM	52	*Abispa ephippium*	80,61		
LEP	75	*Saturnia boisduvalii*	80,62		
COL	21	*Sphaerius* sp.	80,68		
LEP	70	*Eriogyna pyretorum*	80,82	**A4**	**20**
DIP	50	*Simosyrphus grandicornis*	80,84		
LEP	74	*Phthonandria atrilineata*	81,02		
LEP	67	*Bombyx mori*	81,32		
LEP	66	*Bombyx mandarina*	81,68		
LEP	72	*Manduca sexta*	81,79		
HYM	55	*Cephus cinctus*	81,95		
DIP	42	*Drosophila melanogaster*	82,16		
LEP	68	*Coreana raphaelis*	82,66		
DIP	48	*Mayetiola destructor*	84,12		
HYM	53	*Apis mellifera ligustica*	84,86		
DIP	49	*Rhopalomyia pomum*	85,15		
HYM	58	*Melipona bicolor*	86,72	**A5**	**4**
HYM	54	*Bombus ignitus*	86,78		
HYM	56	*Diadegma semiclausum*	87,41		

ORD, order; **N**; reference number in Figure 3; **A+T%**, A+T content expressed in percent; **CL** cluster name; **n**, number of taxa in the cluster. COL, Coleoptera; DIP, Diptera; HYM, Hymenoptera; MCP, Mecoptera; MEG, Megaloptera; NEU, Neuroptera; RPH, Raphidioptera. A1 (65.00 < AT% < 70.00); A2 (70.00 < AT% < 75.00); A3 (75.00 < AT% < 80.00); A4 (80.00 < AT% < 85.00); A5 (85.00 < AT% < 90.00)

Supporting Table S1b. Clusters of AT-skew

ORD	N	TAXON	AT-skew	CL	n
LEP	68	*Coreana raphaelis*	-0,047476104		
LEP	70	*Eriogyna pyretorum*	-0,030596593		
NEU	79	*Polystoechotes punctatus*	-0,028747433		
LEP	75	*Saturnia boisduvalii*	-0,024063307		
LEP	61	*Acraea issoria*	-0,023439428		
LEP	64	*Antheraea yamamai*	-0,022168088		
LEP	63	*Antheraea pernyi*	-0,021477801	**B1**	**13**
HYM	52	*Abispa ephippium*	-0,018660812		
MCP	84	*Neopanorpa pulchra*	-0,013994267		
MEG	81	*Protohermes concolorus*	-0,011148087		
LEP	72	*Manduca sexta*	-0,00535855		
DIP	50	*Simosyrphus grandicornis*	-0,003832005		
LEP	62	*Adoxophyes honmai*	-0,001031337		
HYM	54	*Bombus ignitus*	0,002874974		
DIP	38	*Cydistomyia duplonotata*	0,003159059		
DIP	46	*Haematobia irritans*	0,004483599		
DIP	45	*Drosophila yakuba*	0,005004369		
COL	20	*Rhopaea magnicornis*	0,005058513		
LEP	74	*Phthonandria atrilineata*	0,006688963		
DIP	26	*Aedes albopictus*	0,007921539		
DIP	44	*Drosophila simulans*	0,008485472		
HYM	56	*Diadegma semiclausum*	0,008674404		
DIP	43	*Drosophila sechellia*	0,009226524		
DIP	37	*Culicoides arakawae*	0,009265859		
DIP	41	*Drosophila mauritiana*	0,009459924		
COL	21	*Sphaerius* sp.	0,010491803		
COL	15	*Psacothea hilaris*	0,011439388		
COL	3	*Anoplophora glabripennis*	0,011658031		
LEP	65	*Artogeia melete*	0,012169882		
DIP	40	*Drosophila litoralis*	0,012446774		
MEG	80	*Corydalus cornutus*	0,014043748		
MEG	82	*Sialis hamata*	0,014561518		
NEU	77	*Ditaxis biseriata*	0,015497366		
HYM	58	*Melipona bicolor*	0,015591269		
DIP	47	*Lucilia sericata*	0,015919192	**B2**	**44**
LEP	71	*Lymantria dispar*	0,016082342		
DIP	42	*Drosophila melanogaster*	0,016775803		
DIP	25	*Aedes aegypti*	0,016949153		
HYM	59	*Orussus occidentalis*	0,016949153		
HYM	53	*Apis mellifera ligustica*	0,018315547		
DIP	35	*Chrysomya putoria*	0,020498889		
COL	23	*Trachypachus holmbergi*	0,020574814		
DIP	34	*Ceratitis capitata*	0,021159748		
LEP	69	*Diatraea saccharalis*	0,021379589		
RPH	83	*Mongoloraphidia harmandi*	0,023027851		
HYM	57	*Evania appendigaster*	0,026703233		
COL	10	*Hydroscapha granulum*	0,02972382		
LEP	73	*Ochrogaster lunifer*	0,030153238		
DIP	27	*Anopheles gambiae*	0,032225579		
HYM	55	*Cephus cinctus*	0,034387028		
DIP	36	*Cochliomyia hominivorax*	0,034493953		
DIP	28	*Anopheles quadrimaculatus A*	0,040649047		
COL	12	*Macrogyrus oblongus*	0,04213851		
COL	5	*Chaetosoma scaritides*	0,04274062		
DIP	39	*Dermatobia hominis*	0,042965989		
COL	8	*Crioceris duodecimpunctata*	0,044226044		
DIP	49	*Rhopalomyia pomum*	0,046801619		
COL	14	*Priasilpha obscura*	0,052358082		
LEP	66	*Bombyx mandarina*	0,054727133		
LEP	67	*Bombyx mori*	0,058721799		
DIP	48	*Mayetiola destructor*	0,061457914		
DIP	33	*Bactrocera philippinensis*	0,065619933		
DIP	29	*Bactrocera carambolae*	0,065698419		
DIP	32	*Bactrocera papayae*	0,066233655		
DIP	30	*Bactrocera dorsalis*	0,067543335	**B3**	**16**
NEU	76	*Ascaloptynx appendiculatus*	0,067677946		
COL	13	*Mordella atrata*	0,069147509		
NEU	78	*Libelloides macaronius*	0,071971617		
COL	9	*Cyphon* sp.	0,072114983		
COL	11	*Lucanus mazama*	0,074399531		
HYM	60	*Vanhornia eucnemidarum*	0,085787452		
DIP	31	*Bactrocera oleae*	0,088455511		
DIP	51	*Trichophthalma punctata*	0,091283912		
COL	18	*Rhagophthalmus lufengensis*	0,103881817		
COL	16	*Pyrocoelia rufa*	0,105884066		
COL	24	*Tribolium castaneum*	0,109100492		
COL	6	*Chauliognathus opacus*	0,112081768	**B4**	**7**
COL	1	*Acmaeodera* sp	0,114296016		
COL	19	*Rhagophthalmus ohbai*	0,118513155		
COL	2	*Adelium* sp	0,136789298		
COL	17	*Pyrophorus divergens*	0,16508844	**B5**	**2**
COL	4	*Apatides fortis*	0,170439904		
COL	7	*Chrysochroa fulgidissima*	0,202640643	**B6**	**2**
COL	22	*Tetraphalerus bruchi*	0,247764034		

ORD, order; **N**; reference number in Figure 3; **CL** cluster name; **n**, number of taxa in the cluster. COL, Coleoptera; DIP, Diptera; HYM, Hymenoptera; MCP, Mecoptera; MEG, Megaloptera; NEU, Neuroptera; RPH, Raphidioptera. B1 (-0.050 < AT-skew ≤ 0.000); B2 (0.000 < AT-skew ≤ 0.050); B3 (0.050 < AT-skew ≤ 0.100); B4 (0.100 < AT-skew ≤ 0.150); B5 (0.150 < AT-skew ≤ 0.200); B6 (0.200 < AT-skew ≤ 0.250).

Additional file 1, Negrisolo et al. 2011

Supporting table S1c. Clusters of GC-skew

ORD	N	TAXON	GC-skew	CL	n	ORD	N	TAXON	GC-skew	CL	n
HYM	52	*Abispa ephippium*	-0,379562044	C1	1	COL	15	*Psacothea hilaris*	-0,212955466		
HYM	57	*Evania appendigaster*	-0,349154254			DIP	25	*Aedes aegypti*	-0,210977702		
HYM	60	*Vanhornia eucnemidarum*	-0,328267477			COL	12	*Macrogyrus oblongus*	-0,208083015		
LEP	73	*Ochrogaster lunifer*	-0,317510854	C2	5	DIP	36	*Cochliomyia hominivorax*	-0,206700892	C4	30
HYM	59	*Orussus occidentalis*	-0,307672027			COL	3	*Anoplophora glabripennis*	-0,206674473		
COL	24	*Tribolium castaneum*	-0,305314654			NEU	78	*Ascaloptynx appendiculatus*	-0,205980923		
COL	4	*Apatides fortis*	-0,29462771			LEP	70	*Eriogyna pyretorum*	-0,204761905		
COL	17	*Pyrophorus divergens*	-0,291514413			COL	5	*Chaetosoma scaritides*	-0,200861273		
HYM	55	*Cephus cinctus*	-0,285550459			HYM	56	*Diadegma semiclausum*	-0,198473282		
DIP	31	*Bactrocera oleae*	-0,28020328			LEP	62	*Adoxophyes honmai*	-0,196486662		
COL	11	*Lucanus mazama*	-0,272328549			COL	18	*Rhagophthalmus lufengensis*	-0,195945946		
HYM	54	*Bombus ignitus*	-0,271974229	C3	14	LEP	74	*Phthonandria atrilineata*	-0,192111527		
HYM	53	*Apis mellifera ligustica*	-0,268686869			DIP	34	*Ceratitis capitata*	-0,185102835		
COL	14	*Priasilpha obscura*	-0,264615385			DIP	40	*Drosophila litoralis*	-0,182654402		
MEG	80	*Corydalus cornutus*	-0,26206196			DIP	28	*Anopheles quadrimaculatus A*	-0,181480423		
LEP	69	*Diatraea saccharalis*	-0,257512116			DIP	26	*Aedes albopictus*	-0,181231672		
COL	13	*Mordella atrata*	-0,255675304			LEP	72	*Manduca sexta*	-0,181174805		
MEG	81	*Protohermes concolorus*	-0,253980684			NEU	79	*Ditaxis biseriata*	-0,179378957		
COL	22	*Tetraphalerus bruchi*	-0,251592972			DIP	38	*Cydistomyia duplonotata*	-0,177126918		
HYM	58	*Melipona bicolor*	-0,251174935			NEU	76	*Libelloides macaronius*	-0,176702863	C5	24
COL	1	*Acmaeodera* sp	-0,249268007			MEG	82	*Sialis hamata*	-0,171149867		
LEP	71	*Lymantria dispar*	-0,247366741			DIP	35	*Chrysomya putoria*	-0,170189702		
DIP	51	*Trichophthalma punctata*	-0,244788007			MCP	84	*Neopanorpa pulchra*	-0,167620605		
COL	20	*Rhopaea magnicornis*	-0,238072965			DIP	47	*Lucilia sericata*	-0,166666667		
COL	7	*Chrysochroa fulgidissima*	-0,237364044			COL	8	*Crioceris duodecimpunctata*	-0,163487738		
DIP	37	*Culicoides arakawae*	-0,236957582			NEU	77	*Polystoechotes punctatus*	-0,161232958		
LEP	61	*Acraea issoria*	-0,235255995			COL	16	*Pyrocoelia rufa*	-0,158472673		
DIP	30	*Bactrocera dorsalis*	-0,228353949			LEP	68	*Coreana raphaelis*	-0,157815443		
COL	9	*Cyphon* sp.	-0,228238866			DIP	27	*Anopheles gambiae*	-0,154046997		
RPH	83	*Mongoloraphidia harmandi*	-0,227791878	C4		COL	23	*Trachypachus holmbergi*	-0,153655514		
DIP	39	*Dermatobia hominis*	-0,226784238			COL	6	*Chauliognathus opacus*	-0,153221126		
DIP	32	*Bactrocera papayae*	-0,22638823			COL	10	*Hydroscapha granulum*	-0,151916184		
COL	19	*Rhagophthalmus ohbai*	-0,225648855			DIP	42	*Drosophila melanogaster*	-0,150488225		
DIP	33	*Bactrocera philippinensis*	-0,224499523			COL	21	*Sphaerius* sp.	-0,148249828		
DIP	29	*Bactrocera carambolae*	-0,223752969			DIP	45	*Drosophila yakuba*	-0,136443149		
COL	2	*Adelium* sp	-0,222222222			DIP	44	*Drosophila simulans*	-0,135249622		
LEP	65	*Artogeia melete*	-0,221822934			DIP	43	*Drosophila sechellia*	-0,135102893	C6	10
LEP	64	*Antheraea yamamai*	-0,219980152			DIP	41	*Drosophila mauritiana*	-0,134892086		
LEP	75	*Saturnia boisduvalii*	-0,217069892			DIP	50	*Simosyrphus grandicornis*	-0,132880698		
LEP	63	*Antheraea pernyi*	-0,216321244			DIP	46	*Haematobia irritans*	-0,124517088		
LEP	67	*Bombyx mori*	-0,216290212			DIP	49	*Rhopalomyia pomum*	-0,123084069		
LEP	66	*Bombyx mandarina*	-0,213159698			DIP	48	*Mayetiola destructor*	-0,110068259		

ORD, order; **N**; reference number in Figure 3; **CL,** cluster name; **n**, number of taxa in the cluster. COL, Coleoptera; DIP, Diptera; HYM, Hymenoptera; MCP, Mecoptera; MEG, Megaloptera; NEU, Neuroptera; RPH, Raphidioptera. C1 (-0.400 < GC-skew < -0.350); C2 (-0.300 < GC-skew < -0.250); C3 (-0.300 < GC-skew < -0.250); C4 (-0.250 < GC-skew < -0.200); C5 (-0.200 < GC-skew < -0.150); C6 (-0.150 ≤ GC-skew < -0.100).
