# Supplementary material for: The mitochondrial genome of the ascalaphid owlfly Libelloides macaronius and comparative evolutionary mitochondriomics of neuropterid insects
Source: BMC Genomics. 2011 May 10;12:221. doi: 10.1186/1471-2164-12-221 (PMC3115881; doi:10.1186/1471-2164-12-221)
Supplement: Additional file 2 — Table S2: A+T%, AT-skew, G+C%, GC-skew, for whole genome α strand; pooled α + β strands PCGs; pooled-α strand PCGs and pooled-β strand PCGs. [file 1471-2164-12-221-S2.PDF]

Additional file 2, Negrisolo et al. 2011

Supporting Table S2. A+T%, AT-skew, G+C%, GC-skew, for whole genome α strand; pooled α + β strands PCGs; pooled-α strand PCGs and pooled-β strand PCGs

	**whole genome α strand**				**pooled α + β strands PCGs**					
Taxon	A+T%	AT-skew	G+C%	GC-skew	CDS	A+T%	AT-skew	G+C%	GC-skew	NLC
Ascaloptynx appendiculatus	75.57	0.06768	24.43	-0.205981	3699	73.91	-0.14289	26.09	-0.021762	1
Ditaxis biseriata	79.79	0.0155	20.21	-0.179379	3705	77.18	-0.13859	22.82	0.015773	3
Libelloides macaronius	74.5	0.07197	25.5	-0.176703	3703	73	-0.11739	27	-0.024341	2
Polystoechotes punctatus	78.96	-0.02875	21.04	-0.161233	3706	77.21	-0.1794	22.79	0.035517	5
Corydalus cornutus	74.9	0.01404	25.1	-0.262062	3702	72.64	-0.17268	27.36	0.001645	1
Protohermes concolorus	75.83	-0.01115	24.17	-0.253981	3706	74.02	-0.16428	25.98	0.004848	0
Sialis hamata	78.32	0.01456	21.67	-0.17115	3708	76.12	-0.15116	23.87	0.022222	1
Mongoloraphidia harmandi	80.31	0.02303	19.69	-0.227792	3675	78.1	-0.14893	21.9	-0.000829	2
**average**	77.27	0.021	22.72	-0.205	3700.50	75.27	-0.152	24.73	0.004	1.87
**standard deviation**	2.17	0.035	2.32	0.039	10.68	2.12	0.020	2.13	0.020	1.55

	**pooled-α strand PCGs**						**pooled-β strand PCGs**					
Taxon	CDS	A+T%	AT-skew	G+C%	GC-skew	NLC	CDS	A+T%	AT-skew	G+C%	GC-skew	NLC
Ascaloptynx appendiculatus	2270	72.29	-0.07008	27.71	-0.176471	5	1429	76.49	-0.25221	23.51	0.267857	8
Ditaxis biseriata	2275	75.49	-0.10171	24.51	-0.105798	6	1430	79.88	-0.19405	20.12	0.251448	12
Libelloides macaronius	2272	71.13	-0.033	28.87	-0.156504	4	1431	75.98	-0.2428	24.02	0.227934	5
Polystoechotes punctatus	2274	75.59	-0.17704	24.41	-0.077477	9	1432	79.77	-0.18296	20.23	0.252014	18
Corydalus cornutus	2271	70.54	-0.13525	29.46	-0.191829	3	1431	75.96	-0.22784	24.04	0.377907	9
Protohermes concolorus	2274	72.1	-0.14698	27.9	-0.17814	3	1432	77.07	-0.18997	22.93	0.358376	12
Sialis hamata	2279	74.64	-0.11581	25.35	-0.109059	3	1429	78.49	-0.20475	21.51	0.26898	8
Mongoloraphidia harmandi	2261	76.05	-0.11365	23.95	-0.168719	6	1414	81.38	-0.20162	18.62	0.344304	12
**average**	2272	73.48	-0.112	26.52	-0.145	4.87	1428.5	78.13	-0.212	21.87	0.294	10.5
**standard deviation**	5.24	2.20	0.045	2.20	0.042	2.10	5.98	2.05	0.026	2.05	0.057	3.93

PCG, protein-coding gene; CDS, number of codons; NLC, number of codon(s) lost in the strand.
