# Supplementary material for: The mitochondrial genome of the ascalaphid owlfly Libelloides macaronius and comparative evolutionary mitochondriomics of neuropterid insects
Source: BMC Genomics. 2011 May 10;12:221. doi: 10.1186/1471-2164-12-221 (PMC3115881; doi:10.1186/1471-2164-12-221)
Supplement: Additional file 3 — Figure S1: Relative Synonymous Codon Usage (RSCU) in neuropterid pooled α-strand protein-coding genes. Codon families are provided on the x axis. Red-colored codon, codon not present in the pooled genes. [file 1471-2164-12-221-S3.PDF]

Additional file 3, Negrisolo et al. 2011

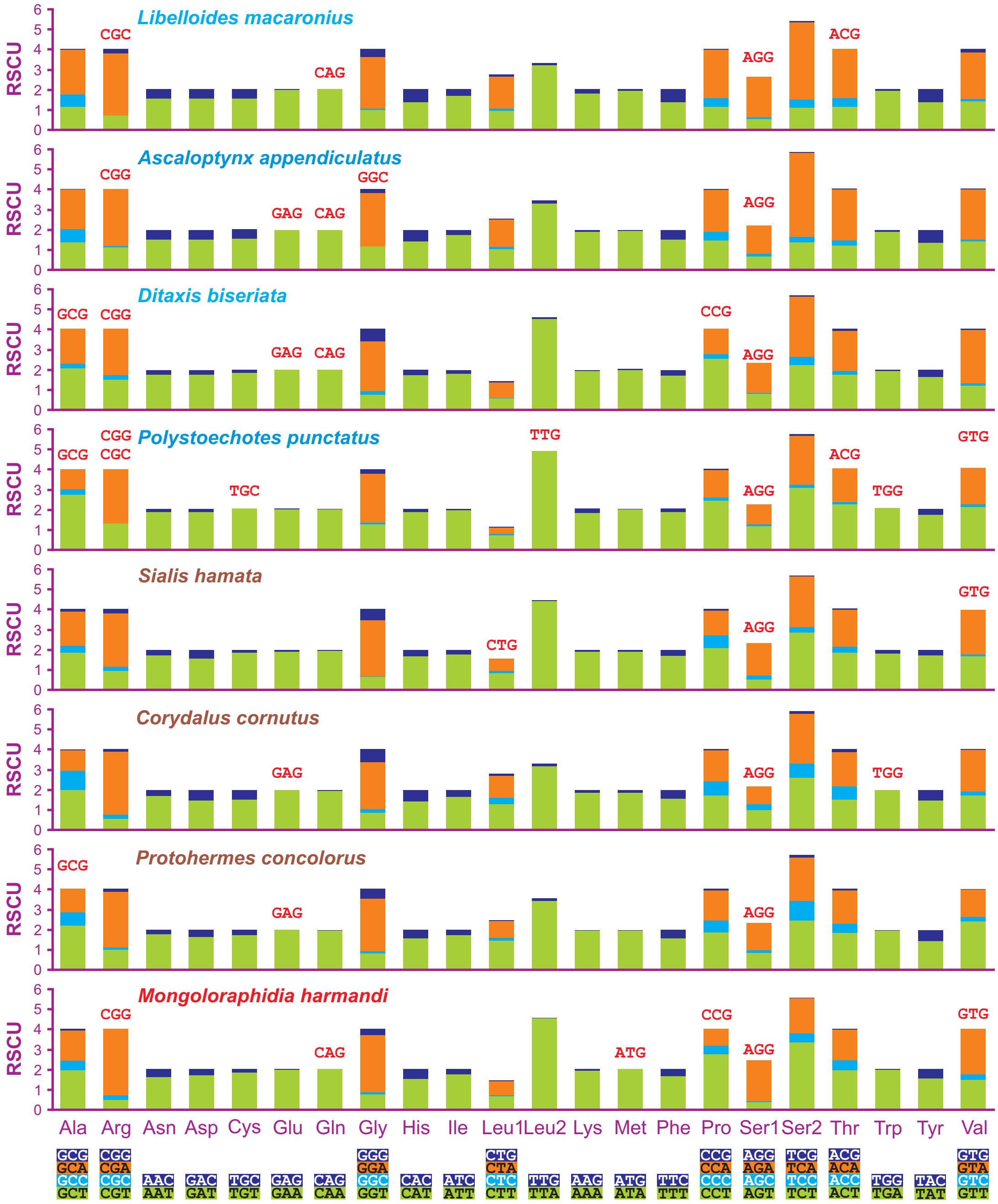

Figure S1. - Relative Synonymous Codon Usage (RSCU) in neuropterid pooled α-strand protein- coding genes .

Codon families are provided on the x axis. Red-colored codon, codon not present in the pooled genes.

Note that the behavior of the families CF$_{Ala}$, CF$_{Pro}$ and CF$_{Val}$ differs from that observed for the pooled-α+β PCGs . In the first two families the adenine is the most common nucleotide in third positions of ascalaphid *A. appendiculatus* and *L. macaronius* while the thymine is the most abundant in all other neuropterids. In CF$_{Val}$ adenine is the most abundant in third positions with exception represented by *P. punctatus* and *P. concolorus* where it is superseded by thymine.
