# Supplementary material for: The mitochondrial genome of the ascalaphid owlfly Libelloides macaronius and comparative evolutionary mitochondriomics of neuropterid insects
Source: BMC Genomics. 2011 May 10;12:221. doi: 10.1186/1471-2164-12-221 (PMC3115881; doi:10.1186/1471-2164-12-221)
Supplement: Additional file 4 — Figure S2: Relative Synonymous Codon Usage (RSCU) in neuropterid pooled β-strand protein-coding genes. Codon families are provided on the x axis. Red-colored codon, codon not present in the pooled genes. [file 1471-2164-12-221-S4.PDF]

Additional file 4, Negrisolo et al. 2011

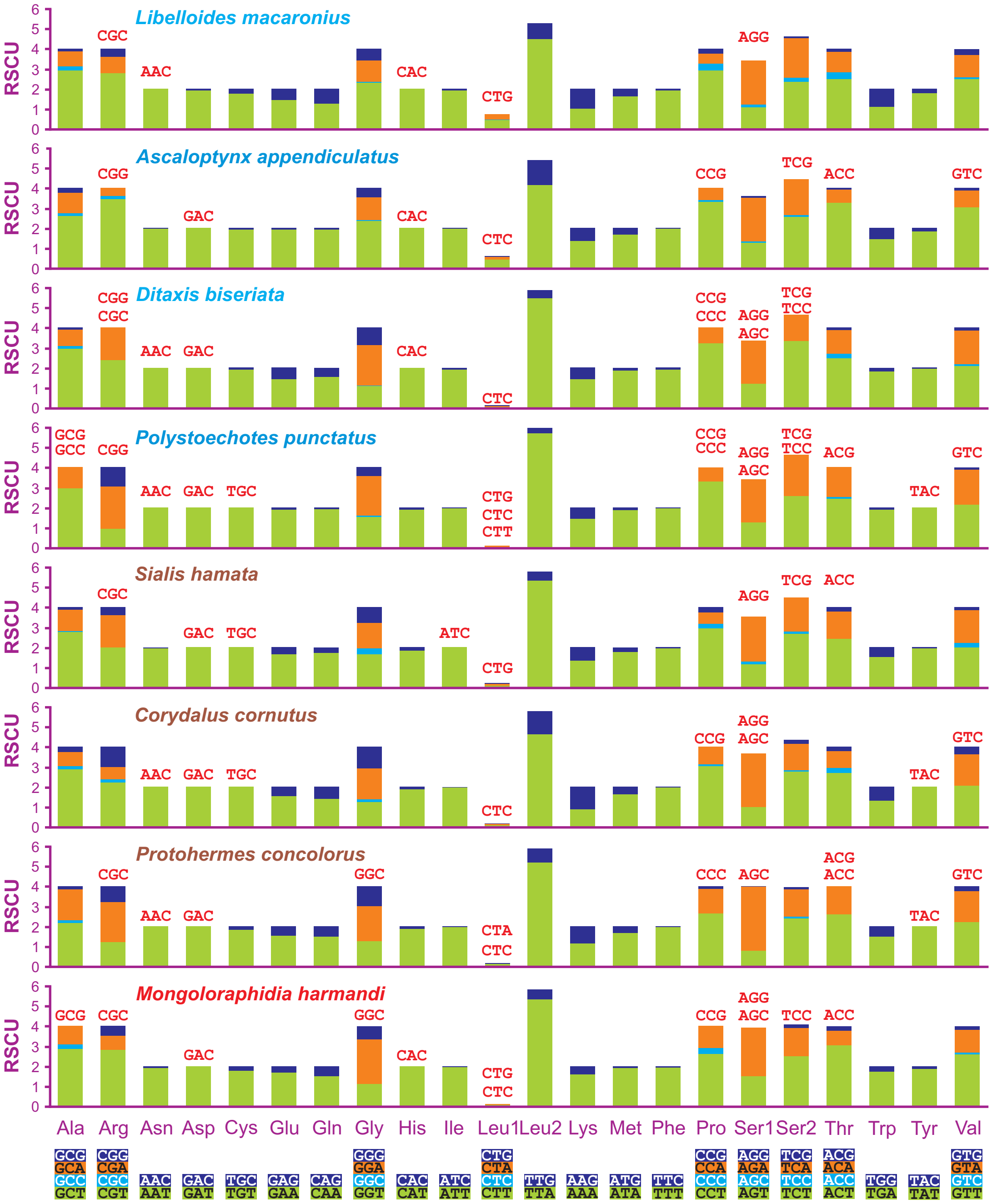

Figure S3. - Relative Synonymous Codon Usage (RSCU) in neuropterid pooled β-strand protein-coding genes.

Codon families are provided on the x axis. Red-colored codon, codon not present in the pooled genes.
Loss of synonymous GC-rich codons is common in fourfold codon familes (CFs*)* (e.g. CF_{Ser1}, CF_{Ser2}). In CF_{Arg} abundance of A vs. T in third positions is very variable ranging from strong bias toward A (e.g. *A. appendiculatus)* up to strong bias toward T (*P. punctatus*). The twofold degenerate CF_{Lys} shows also a variable pattern with G preferred to A in *C. cornutus*, G equaling A (*L. macaronius*) or A favored over G (other species).
