# Supplementary material for: The mitochondrial genome of the ascalaphid owlfly Libelloides macaronius and comparative evolutionary mitochondriomics of neuropterid insects
Source: BMC Genomics. 2011 May 10;12:221. doi: 10.1186/1471-2164-12-221 (PMC3115881; doi:10.1186/1471-2164-12-221)
Supplement: Additional file 5 — Table S3: Summary of multiple alignments of tRNA families in neuropterid mtDNAs. [file 1471-2164-12-221-S5.PDF]

Supporting Table S3. Summary of multiple alignments of tRNA genes in neuropterid mtDNAs.

**ALN**	**amino acid**	**alignment length**	**identical positions**	**%INUC**
trnA	Alanine	68	39	57.35
trnC	Cysteine	70	27	38.57
trnD	Aspartate	70	40	51.43
trnE	Glutamate	70	49	70.00
trnF	Phenylalanine	68	31	45.59
trnG	Glycine	66	51	77.27
trnH	Histidine	70	40	57.14
trnI	Isoleucine	68	40	58.82
trnK	Lysine	71	54	76.06
trnL1	Leucine (CUN)	68	39	57.35
trnL2	Leucine (UUR)	66	45	68.18
trnM	Methionine	70	50	71.43
trnN	Asparagine	68	49	72.02
trnP	Proline	69	42	60.87
trnQ	Glutamine	69	45	65.28
trnR	Arginine	69	35	50.72
trnS1	Serine (AGN)	68	41	60.29
trnS2	Serine (UCN)	67	43	64.18
trnT	Threonine	65	41	63.08
trnV	Valine	73	38	52.05
trnW	Tryptophan	68	47	69.18
trnY	Tyrosine	72	47	65.28

ALN, alignment name; %INUC, percent of identical nucleotides
