# Supplementary material for: The mitochondrial genome of the ascalaphid owlfly Libelloides macaronius and comparative evolutionary mitochondriomics of neuropterid insects
Source: BMC Genomics. 2011 May 10;12:221. doi: 10.1186/1471-2164-12-221 (PMC3115881; doi:10.1186/1471-2164-12-221)
Supplement: Additional file 6 — Figure S3: Domain I of rrnL in neuropterid species and in Drosophila melanogaster. Green background, conserved nucleotide in the pair-wise alignment with L. macaronius. [file 1471-2164-12-221-S6.PDF]

Additional file 6, Negrisolo et al. 2011

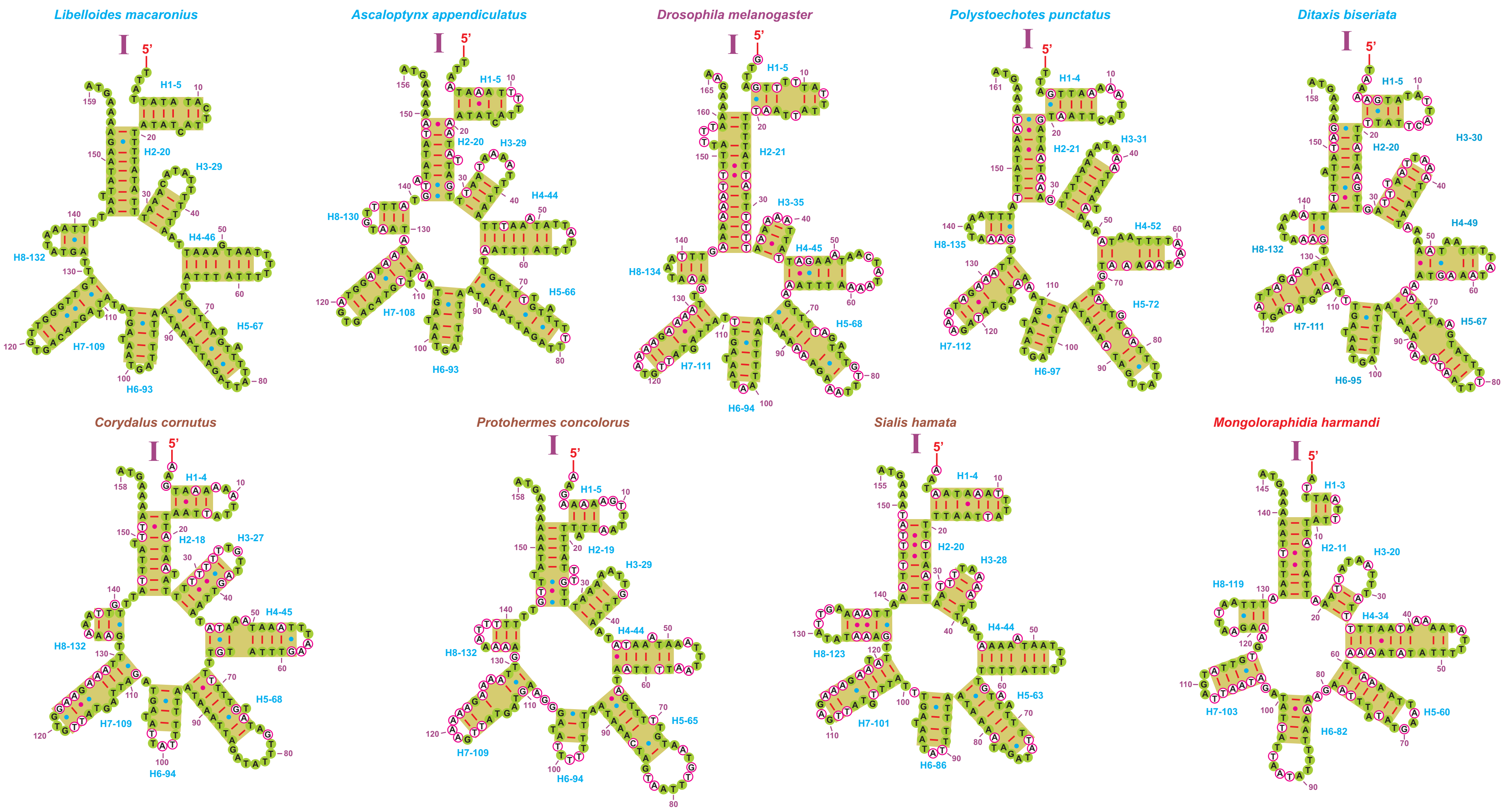

Figure S3. - Domain I of *rrnL* in neuropterid species and in *Drosophila melanogaster*.

Green background, conserved nucleotide in the pair-wise alignment with *L. macaronius*.
